# Aging‐Associated Vacuolation of Multi‐Ciliated Cells in the Distal Mouse Oviduct Reflects Unique Cell Identity and Luminal Microenvironment

**DOI:** 10.1111/acel.70051

**Published:** 2025-05-01

**Authors:** Keerthana Harwalkar, Nobuko Yamanaka, Alain S. Pacis, Selina Zhao, Katie Teng, Warwick Pitman, Mitaali Taskar, Vera Lynn, Alex Frances Thornton, Matthew J. Ford, Yojiro Yamanaka

**Affiliations:** ^1^ Department of Human Genetics McGill University Montreal Quebec Canada; ^2^ Rosalind and Morris Goodman Cancer Institute (GCI) McGill University Montreal Quebec Canada; ^3^ McGill's Integrated Core of Animal Modeling (MICAM) McGill University Montreal Quebec Canada; ^4^ Canadian Centre for Computational Genomics (C3G), McGill Genome Centre McGill University Montreal Quebec Canada

**Keywords:** aging, chronic stimulation, hypoxia, mouse, steroid control of aging

## Abstract

The female reproductive organs present with the earliest aging characteristics, such as a decline in fertility and estrous cyclicity. While age‐related changes in the ovary are well documented, it is unclear if any age‐associated changes occur in the other female reproductive organs, such as the oviduct/Fallopian tube. At the distal end of aged oviducts in mice, we found vacuolated multi‐ciliated cells (MCCs) with a severely apically displaced and deformed nucleus. This phenotype was unique to the distal oviduct epithelium—the infundibulum (INF) and ampulla (AMP). Ovariectomy did not affect the timeline of MCC vacuolation, suggesting little involvement of ovulation and hormonal regulation. MCC vacuolation was induced in hypoxia or hydroxyurea treatments in in vitro organotypic culture of all oviduct regions, not limited to the INF/AMP epithelium. This suggests a high oxygen demand in MCCs, compared to other cell types, and a uniquely stressed INF/AMP epithelial microenvironment in vivo. We found that the blood circulation of INF/AMP depended on the ovarian artery, different from the rest of the oviduct epithelium, and its circulation declined along with ovarian activities. We conclude that a decline in local blood circulation and distinct cellular identity of the INF/AMP epithelium caused age‐associated MCC vacuolation, reflecting its mild, chronically stressed microenvironment.

## Graphical abstract

Multi‐ciliated cells in the infundibulum and ampulla (INF/AMP) epithelium are vacuolated in aging. Unique cellular susceptibility of the INF/AMP epithelial population and aging‐associated decline in ovarian artery circulation, which supports the ovary and INF/AMP, contribute to this region‐specific vacuolation phenotype, as a consequence of a mildly stressed microenvironment.

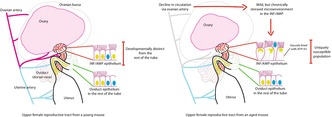

## 1 Introduction

The oviduct is the tube that extends from the uterus to the ovary, with four regions demarcated by their mucosal fold morphologies, epithelial cell transcription factor expression, and proportion/distribution of multi‐ciliated cells (MCCs). These regions are the infundibulum/ampulla (INF/AMP), ampulla‐isthmus junction (AIJ), isthmus (ISM), and uterotubal junction (UTJ) from distal to proximal, relative to the uterus (Harwalkar et al. 2021; Harwalkar and Yamanaka 2021). Gamete and preimplantation embryo movement occurs in the oviduct, aided by the mucosal epithelium (Shi et al. 2011; Yuan et al. 2021) and smooth muscle contractions (Ishikawa et al. 2016). These functions are controlled by hormonal regulation, via the hypothalamic–pituitary–ovary axis, in a cyclic manner over the course of 4–5 days, called the estrous cycle in mice. Peak estrous cyclicity is noted at around 5 months of age in single housed, unmated, inbred C57BL/6J mice, and a decline in this cyclicity occurs from ~9 months. The initial phase of decline that occurs at 10–12 months of age is characterized by a progressive lengthening of estrous cycles (Nelson et al. 1982). Alongside this loss of estrous cyclicity, a decline in oocyte quality, impaired decidualization, uterine fibrosis (Nelson et al. 2013; Wu et al. 2023), prolongation of gestation/labour, and decreased litter size (Patel et al. 2017) are noted.

Although classified as a type of ovarian cancer, High Grade Serous Ovarian Carcinomas (HGSCs) originate from the distal fimbrial region of the fallopian tubes (Medeiros et al. 2006; Piek et al. 2001). The frequency of ovulation across the reproductive lifespan is considered a risk factor because of repetitive exposures to inflammatory cytokines and reactive oxygen species (ROS) that can induce mutations in the adjacent fimbrial epithelium (Karst and Drapkin 2010). On the other hand, the distal INF/AMP epithelium is developmentally and transcriptionally distinct from the rest of the oviduct epithelia (Ford et al. 2021; Harwalkar et al. 2021). Therefore, this raises the possibility that the INF/AMP epithelial cells are uniquely susceptible to malignant transformation, a concept known as cellular pliancy (Puisieux et al. 2018). HGSCs are diagnosed later in life, in postmenopausal individuals typically over 50 years of age (Quirk et al. 2005), suggesting that aging may provide a unique permissive environment for HGSC initiation and/or progression.

In this study, we identified MCC‐specific vacuolation in the INF/AMP epithelium of mice at around 7 months of age. The size and frequency of these MCC vacuoles increased with age. Ovariectomy did not change the timeline of vacuolation, suggesting no association with repetitive ovulation or hormonal regulation. Hypoxia and hydroxyurea (HU) treatments in in vitro organotypic cultures induced vacuolation in MCCs of all oviduct regions, not limited to the INF/AMP epithelium. This confirms the general high energy demand of MCCs but simultaneously suggests a unique INF/AMP microenvironment in vivo. We realized that the INF/AMP blood circulation was coupled to that of the ovary via the ovarian artery and demonstrated that blood circulation into the ovary and INF/AMP was reduced in aged females. Thus, we conclude that MCC vacuolation in the INF/AMP epithelium of aged females is primarily due to the constant high‐energy demand of MCCs compared to other cell types. MCC vacuolation unique to the INF/AMP epithelium was caused by its distinct cellular property and a mild, chronically stressed INF/AMP microenvironment due to unique circulation patterns. We propose that this could contribute to age‐associated vulnerability to HGSC.

## 2 Results

### 2.1 Epithelium‐Lined Diverticula Along the Aged Mouse Oviduct

**FIGURE 1:**
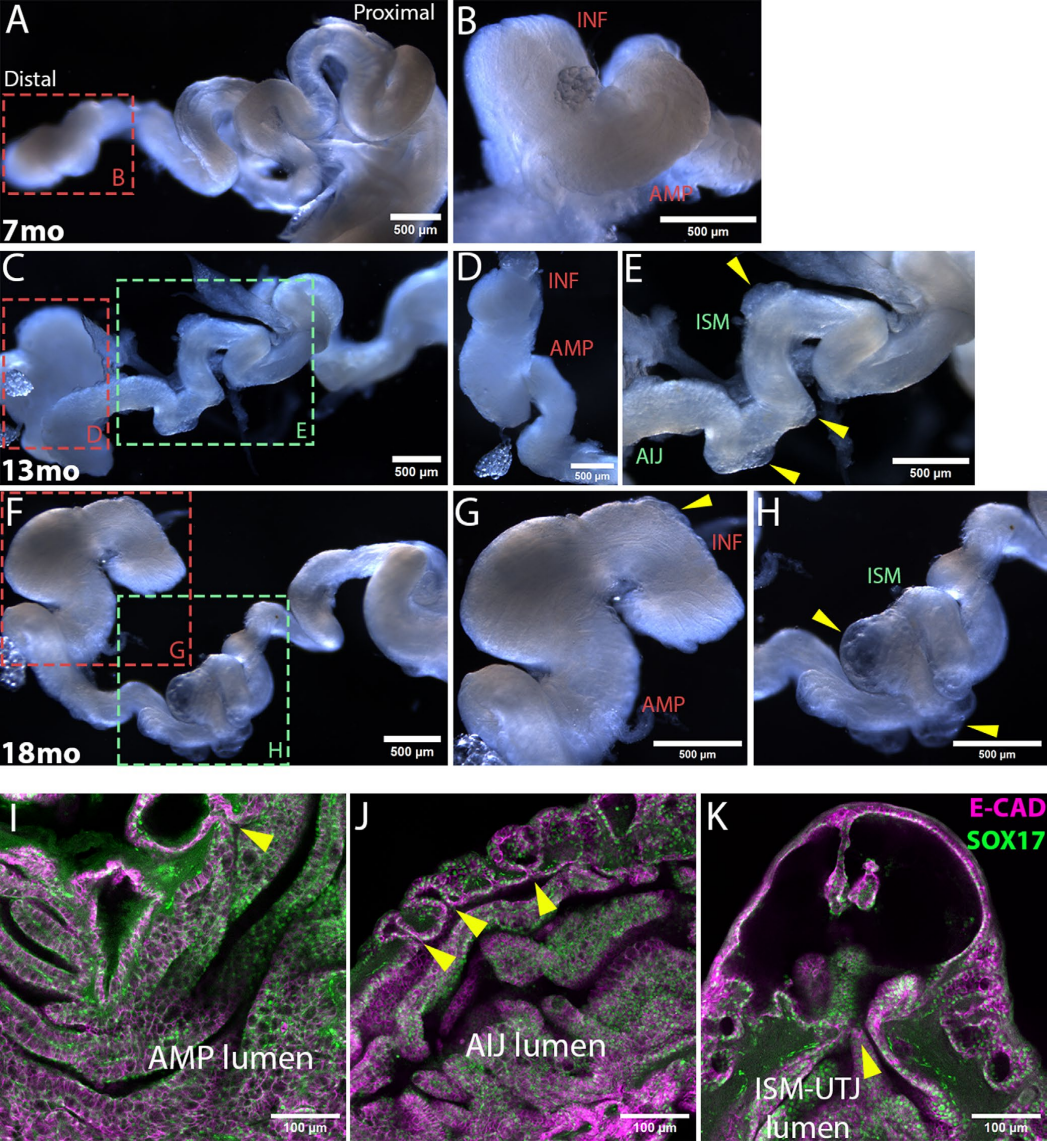
Epithelial outpouching along the aged mouse oviduct. (A–H) Whole‐mount images of an uncoiled oviduct dissected from a 7mo (A and B; *N* = 5), 13mo (B–D; *N* = 5) and 18mo (E–H; *N* = 5) mouse showing outpouching (yellow arrows in E, G, H). Aged 13mo and 18mo oviducts were stiffer and less translucent than 7mo oviducts. (I–K) 3D confocal images of an 18mo AMP (I), AIJ (J), and ISM‐UTJ (K) regions showing E‐CAD and SOX17‐expressing epithelial cells lined the diverticula and connected into the oviduct lumen (yellow arrows in I–K).

To investigate any age‐related structural changes in the mouse oviduct, we examined whole‐mount and 3D confocal images of oviducts isolated from 7‐month‐old (7mo), 13‐month‐old (13mo), and 18‐month‐old (18mo) females. A 7mo oviducts did not present with any obvious structural changes (Figure 1A,B), relative to 2‐4mo oviducts (Harwalkar et al. 2021). Outpouching of the smooth muscle layer was noted in 13mo oviducts, predominantly in the proximal AIJ and ISM regions (Figure 1C,E), but not evident in the distal INF/AMP (Figure 1D). In oviducts isolated from 18mo mice, the distal INF/AMP region presented with outpouching of the smooth muscle (Figure 1G), similar to those noted in the proximal oviducts of 13mo mice (Figure 1E). Large outpouched cysts were restricted to the proximal oviduct (Figure 1F,H). Interestingly, these cysts observed throughout the tube in 18mo mice were lined with E‐CAD+ epithelium that connected into the oviduct lumen (Figure 1I–K), indicating that these structures are diverticula. Indeed, the outpouched epithelium that faced the lumen was SOX17+ (Figure 1I–K), confirming that it originated from the oviduct epithelium (Harwalkar et al. 2021). It is likely caused by tears in the smooth muscle layer, potentially due to the stiffened tube structure (Winkler et al. 2024) and estrous cycle‐associated changes in luminal pressure. Age‐associated fibrosis, muscle layer thickness, and continued peristaltic movements of the oviduct could contribute to the formation of diverticula; in particular, the large diverticula observed in the ISM and UTJ.

### 2.2 Large Cytoplasmic Vacuole With an Apically Displaced, Crescent‐Shaped Deformed Nucleus Was Uniquely Noted in INF/AMP MCCs of the Aged Oviduct

**FIGURE 2:**
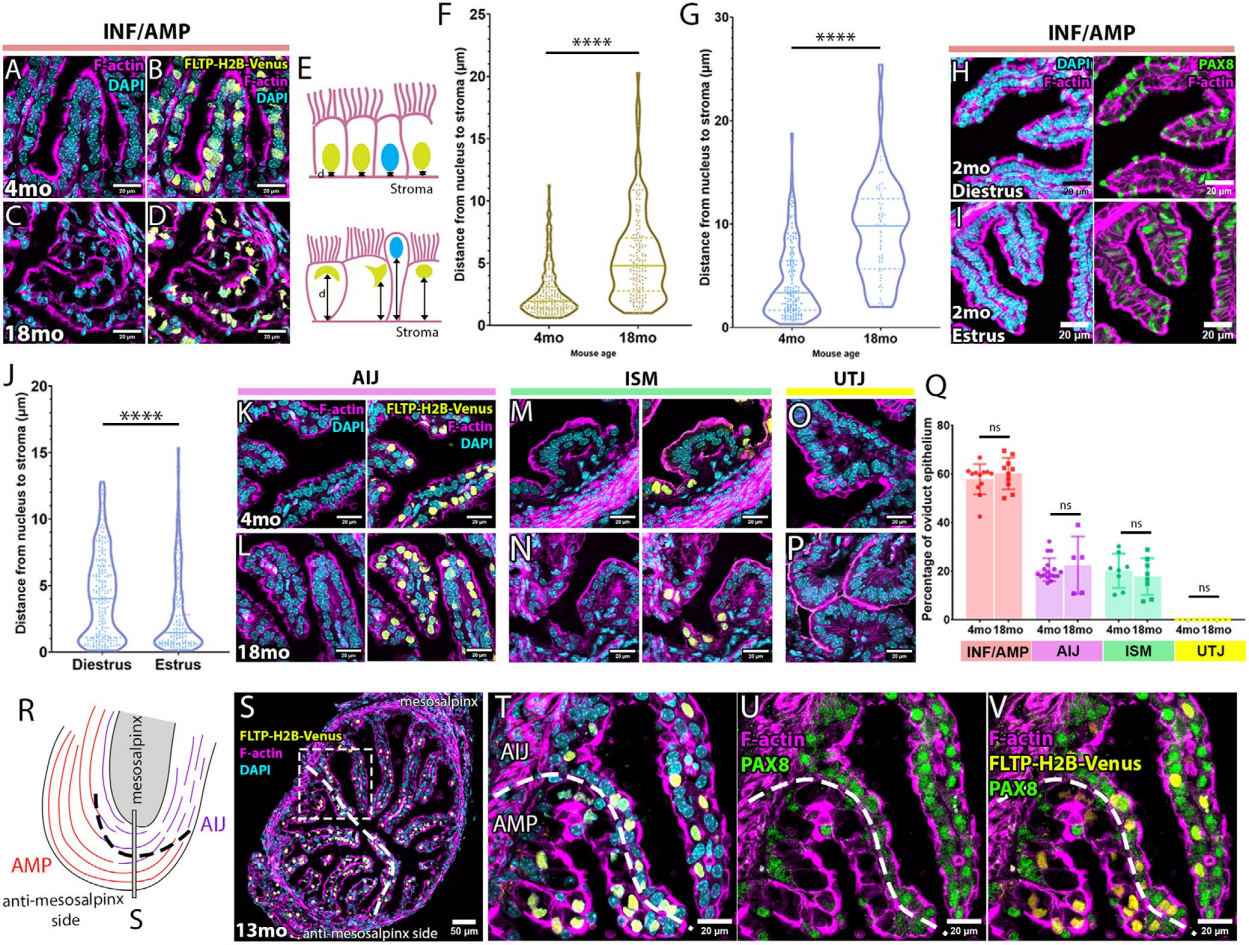
Aging‐associated MCC vacuolation and accompanied apical displacement of the nucleus with nuclear anomaly are restricted to the INF/AMP epithelium. (A–D) Morphology of epithelial cells lining the INF/AMP mucosal folds in oviducts dissected from 4mo (A, B) and 18mo (C, D) mice. DAPI staining visualized the nuclear shape of epithelial cells, and FLTP‐H2B‐Venus marked MCC nuclei. In the 18mo INF/AMP epithelium, MCC nuclei were apically displaced (D), relative to 4mo INF/AMP (B). (E) Illustration showing measurement of nuclear apical displacement in 4mo and 18mo INF/AMP epithelium. (F) Measurements of the distance between MCC nuclei to stroma in the INF/AMP region of 4mo (mean ± SD, 2.606 ± 1.958 μm; *N* = 3) and 18mo (5.482 ± 3.541 μm; *N* = 3) oviducts. (G) Measurements of the distance between SC nuclei to stroma in the INF/AMP region of 4mo (4.415 ± 3.45 μm; *N* = 3) and 18mo (9.324 ± 4.559 μm; *N* = 3) oviducts. Each point represents a measurement/cell. The continuous line represents the median, while the dotted lines show the quartiles. ****p < 0.0001. (H, I) Morphology of the INF/AMP epithelium of young 2mo mice in the estrus stage. PAX8+ SCs were aligned with other epithelial cells in the epithelium during estrus (I), while they apically protruded from the epithelium during diestrus (H). (J) Measurements of the distance of SC nuclei to stroma in the INF/AMP region of mice in the estrus stage (2.791 ± 3.007 μm; *N* = 3) and in the diestrus stage (4.28 ± 3.219 μm; *N* = 3) oviducts. (K–P) Morphology of epithelial cells lining the AIJ (K, L), ISM (M, N) and UTJ (O, P) mucosal folds in oviducts dissected from 4mo (K, M, O) and 18mo (L, N, P) mice. (Q) Percentage of MCCs in the INF/AMP, AIJ, ISM, and UTJ of 4mo and 18mo oviducts, ns = *p* > 0.05. Error bars indicate standard deviation. Datapoints/replicates are represented by circles (4mo) or squares (18mo). (R) Illustration of the beveled, sharp boundary of mucosal fold morphology, cell subtype, and change in proportion of MCCs between turns 2 and 3. (S) Transverse section of the sharp boundary between turns 2 and 3, as illustrated in (R). Sudden drop in MCC proportion on the mesosalpinx side, characteristic of the AMP‐AIJ boundary. (T–V) Apical displacement of nuclei restricted to the AMP, but not evident in the AIJ (T). Change in PAX8 expression in AMP and AIJ epithelium (U), with MCCs being PAX8‐ in the AMP but PAX8+ in the AIJ (V), confirming distinct cell subtypes at this boundary.

To investigate any age‐related changes at the cellular level, we examined transverse sections of oviducts isolated from 4‐month‐old (4mo) and 18mo females from four distinct regions: INF/AMP, AIJ, ISM, and UTJ (Figure 2A–D,K–P). In the 4mo INF/AMP epithelium, the nucleus of epithelial cells was round or oval shaped and positioned basally (Figure 2A,B). Many epithelial cells in the 18mo INF/AMP epithelium carried a large cytoplasmic vacuole, with an apically displaced nucleus that was often crescent‐shaped due to the vacuole (Figure 2C). Nuclear FLTP‐H2B‐Venus expression (Gegg et al. 2014), previously shown to mark MCCs along the oviduct (Harwalkar et al. 2021), indicated that all vacuolated cells were MCCs (Figure 2D). We measured the distance between the base of the nucleus and the stroma (Figure 2E) to quantify the severity of nuclear displacement and found that the apical displacement of MCC nuclei in 18mo mice was significantly larger than in 4mo mice (Figure 2F). The nucleus of secretory cells (SCs) was also apically displaced and protruded from the epithelial monolayer in 18mo oviducts, although their nuclear shape remained round or oval (Figure 2C,D). Similar to MCCs, the apical displacement of SC nuclei at 18mo was significantly larger than at 4mo (Figure 2G). It is reported that SC protrusion from the epithelial monolayer is observed during the female hormonal cycle (Komatsu and Fujita 1978). In agreement with this, we found that protrusion of SC nuclei, marked by PAX8, was prevalent during the diestrus stage (Figure 2H,J), as compared to the estrus stage (Figure 2I,J) in young females, suggesting that SC protrusion was not exclusive to aging.

Interestingly, the MCC vacuolation and apically displaced crescent‐shaped nuclei were observed in the INF/AMP epithelium (Figure 2A–D) but not in the other regions of the oviduct. The nuclear shape in the AIJ, ISM, and UTJ epithelium was generally round or oval, with no sign of cytoplasmic vacuolation (Figure 2K–P). There was no apparent difference in the distribution pattern (Figure 2A–D,K–P) and proportion (Figure 2Q) of MCCs between 4mo and 18mo oviducts. We concluded that MCC vacuolation and apical displacement of the nucleus were aging phenotypes unique to the INF/AMP epithelium.

We previously established that there is a sharp boundary between each distinct region of the oviduct, visualized by changes in transcription factor expression, MCC proportion/distribution, and mucosal fold morphology (Harwalkar et al. 2021). At the beveled AMP‐AIJ boundary, located between oviduct coiling/turning points 2 and 3, the AMP epithelium occupied the anti‐mesosalpinx side recognized by a higher proportion of MCCs, while the AIJ epithelium began on the mesosalpinx side (Figure 2R,S). We found that only AMP MCCs, indicated by lack of PAX8 expression, had a large cytoplasmic vacuole with an apically displaced, crescent‐shaped nucleus, while the AIJ MCCs did not show any apparent morphological abnormality, despite identical proximity to the ovary (Figure 2T–V). This suggests that the aging‐associated MCC vacuolation in the INF/AMP epithelium is linked with its unique cell property rather than physical proximity to the ovary.

### 2.3 Progressive Increase in Size, Frequency of MCC Vacuolation and Apical Nuclear Displacement in the INF/AMP Epithelium After 6 Months

**FIGURE 3:**
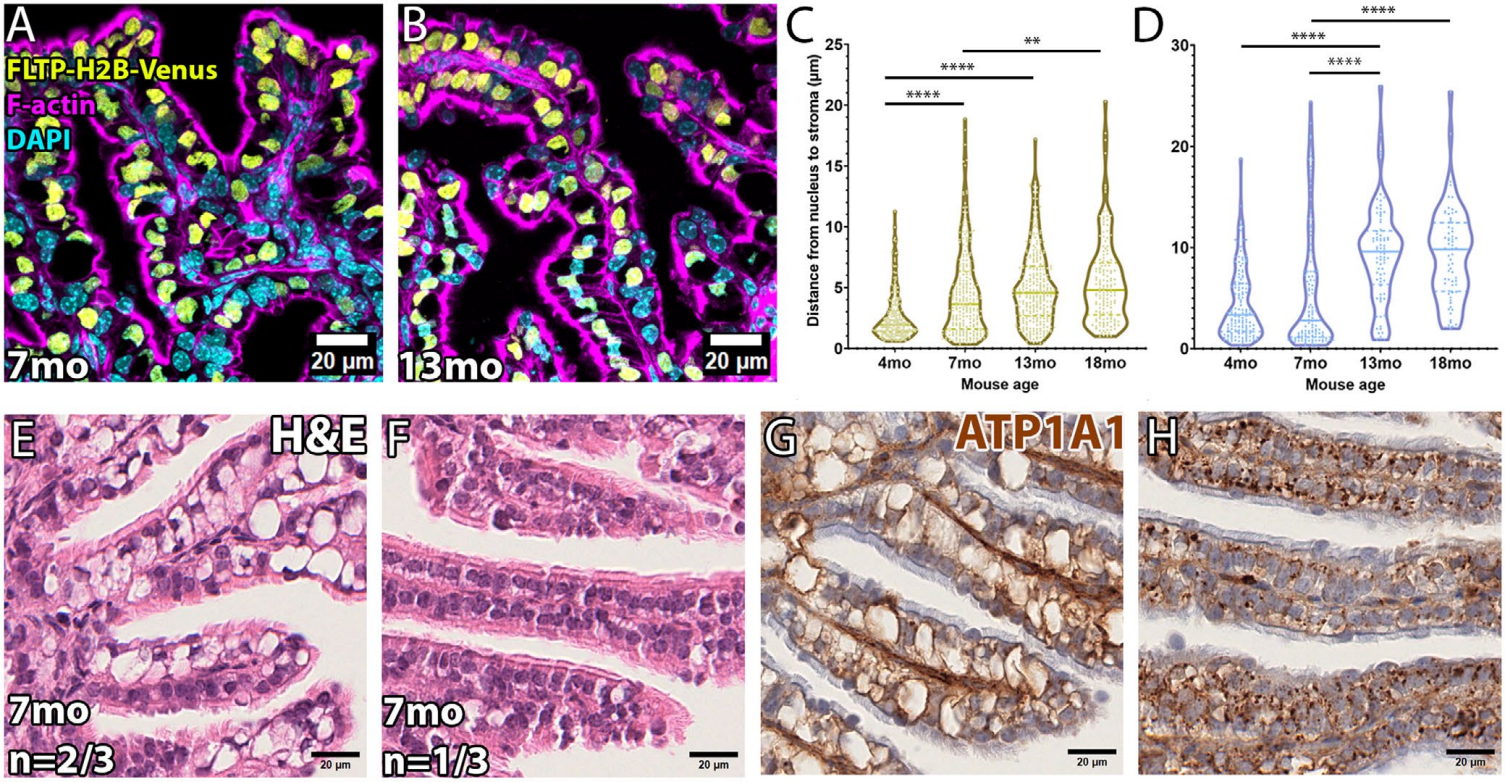
Progressive apical displacement of the nucleus and large vacuole with aqueous fluid in INF/AMP MCCs. (A, B) Morphology of epithelial cells lining the INF/AMP mucosal folds in oviducts dissected from 7mo (A) and 13mo (B) mice. SC nuclei and deformed MCC crescent‐shaped nuclei were apically displaced. (C) Measurements of the distance of MCC nuclei to stroma in the INF/AMP region of 4mo, 7mo (4.476 ± 3.543 μm; *N* = 3), 13mo (5.014 ± 3.085 μm; *N* = 3), and 18mo oviducts. (D) Measurements of the distance of SC nuclei to stroma in the INF/AMP region of 4mo, 7mo (5.628 ± 6.045 μm; *N* = 3), 13mo (9.285 ± 4.790 μm; *N* = 3), and 18mo oviducts. Each point represents a measurement/cell. The continuous line represents the median, while the dotted lines show the quartiles. ****p < 0.0001, **p < 0.01. (E, F) H&E staining of the 7mo INF/AMP region. Two phenotypes were noted: (1) exaggerated/large, empty cytoplasmic vacuoles and apically displaced nuclei (E) and (2) small/indiscernible empty spaces/vacuoles and nuclei located in the centre of the cells (F). (G, H) ATP1A1 expression in 7mo INF/AMP region. Large cytoplasmic vacuoles were lined with ATP1A1 (G), while MCCs with small/indiscernible vacuoles contained ATP1A1+ punctae (H).

To determine the timeline of this age‐related phenotype in the distal INF/AMP epithelium, we examined 7mo and 13mo oviducts. Both 7mo and 13mo INF/AMP epithelium had varying proportions of apically displaced MCC nuclei (Figure S1A), with most SC nuclei protruding from the epithelial monolayer (Figure 3A–B). The position of the nucleus in INF/AMP MCCs was progressively apically displaced from 4mo to 18mo (Figure 3C). Apical displacement of SC nuclei at 13mo was significantly larger than 4mo and 7mo, but not significantly different from 18mo (Figure 3D), due to SC body protrusion from the epithelial monolayer at 13mo and 18mo (Figure 3A–B). We found individual female variation in 7mo oviducts: (1) some individuals exhibited exaggerated apical displacement of MCC nuclei and large, empty cytoplasmic spaces in many cells (Figure 3E; *N* = 2/3), similar to that noted in 13mo or 18mo mice, and (2) an individual presented with low‐to‐moderate apical displacement with small cytoplasmic spaces (Figure 3F; *N* = 1/3).

A vacuole was located at the basal side of MCCs, clearly distinct from secretory vesicles typically located at the apical side. The vacuole appeared to cause apical displacement and crescent‐shaped deformation of the nucleus. Hematoxylin and eosin (H&E) staining showed empty cytoplasmic spaces, ruling out the possibility of diluted cytoplasm, protein inclusions, or nucleic acid accumulation (Figure 3E,F). In agreement with this, a classic TEM study of the AMP epithelium in 7‐ to 24‐month‐old mice shows MCCs with large vacuoles containing heterogeneously dense materials, and no obvious changes in other organelles including cilia (Komatsu and Fujita 1978). We found that the large cytoplasmic vacuole was lined with sodium/potassium‐transporting ATPase subunit alpha‐1 (ATP1A1; Figure 3G) and did not show lipid or glycogen accumulation (Figure S1B–D), suggesting aqueous/fluid accumulation in INF/AMP MCCs. Cytoplasmic, punctate ATP1A1 localization surrounded the small vacuoles in MCCs with low‐to‐moderate apical displacement (Figure 3H). Cytoplasmic ATP1A1 was not observed in epithelium of the proximal regions (Figure S1E–G). Occasional punctate ATP1A1 was noted in the INF/AMP of 3mo mice (Figure S1H).

### 2.4 INF/AMP MCC Vacuolation Was Independent of Ovulation and Estrous Cycling

The INF/AMP region is located directly adjacent to the ovary, a reproductive organ that undergoes ovulation every 4–5 days in mice (Bertolin and Murphy 2014). We considered two possibilities: (1) the distal oviduct is exposed to events associated with ovulation, including exposure to oxidative stress, inflammation‐like response, and production of reactive oxygen species (ROS) that induce repetitive stress in the adjacent INF/AMP epithelium (Murdoch and Martinchick 2004), and (2) the other possibility is estrous cycle‐associated events like epithelial cell turnover. It is known that the INF/AMP epithelium shows fluctuation in the proportion of SCs and MCCs in response to hormonal regulation (Ford et al. 2023). Decline in estrous cyclicity occurs by ~9 months in inbred mice (Felicio et al. 1984), characterized by a progressive lengthening of estrous cycles followed by cyclic cessation and anestrus by 11–16 months of age. Thus, age‐associated lack of hormonal cycling may contribute to MCC vacuolation due to reduced cell turnover.

**FIGURE 4:**
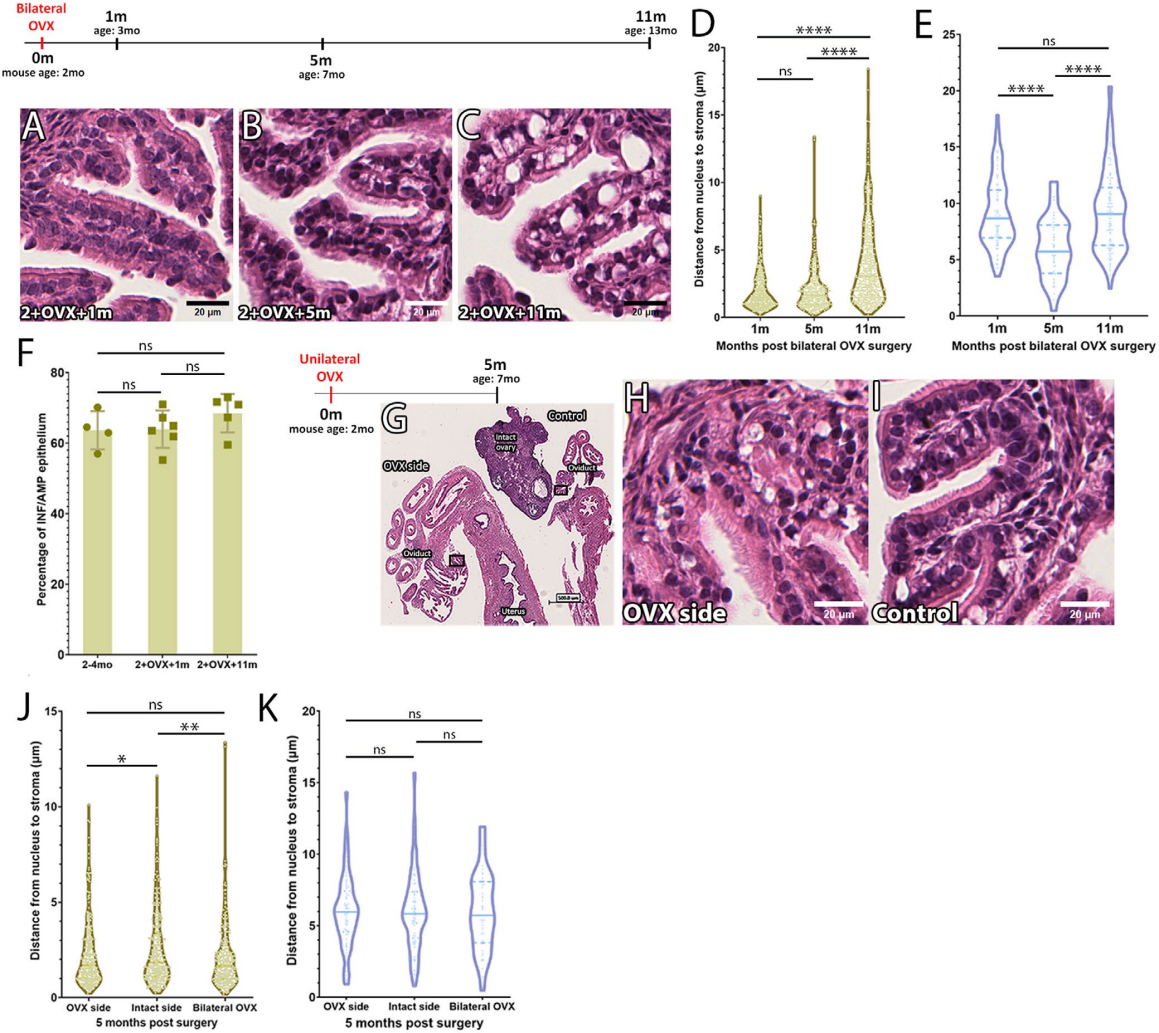
Timeline of the INF/AMP MCC vacuolation is unaffected regardless of loss of ovulation and estrous cycling. (A–C) H&E staining of the INF/AMP region in oviducts dissected from mice 1 month (A), 5 months (B), and 11 months (C) following bilateral OVX. MCC vacuolation is observed in the INF/AMP epithelium of mice 5 and 11 months post bilateral OVX. (D) Measurements of the distance of MCC nuclei to stroma in the INF/AMP epithelium of mice 1 month (1.937 ± 1.402 μm; *N* = 3), 5 months (2.077 ± 1.656 μm; *N* = 3), and 11 months (3.977 ± 2.977 μm; *N* = 4) post bilateral OVX. (E) Measurements of the distance of SC nuclei to stroma in the INF/AMP epithelium of mice 1 month (9.124 ± 3.146 μm), 5 months (5.994 ± 2.682 μm), and 11 months (9.334 ± 3.694 μm) post bilateral OVX. Changes in apical displacement of SC nuclei indicated corresponded with changes in the height of the epithelium. (F) Percentage of MCCs in the INF/AMP epithelium of oviducts from non‐OVX 2–4mo mice, and OVX mice dissected at 1 and 11 months post surgery, ^ns^ *p* > 0.05. Error bars indicate standard deviation. Datapoints/replicates are represented by circles (non‐OVX) or squares (OVX). (G–I) H&E staining of the INF/AMP region in oviducts dissected from mice 5 months following unilateral OVX. MCC vacuolation was noted in both the ovariectomized/OVX side (H) and the side with the ovary intact/intact side (I). (J) Measurements of the distance from MCC nuclei to stroma in the INF/AMP region of mice 5 m following unilateral OVX, comparing distances on the OVX side (2.220 ± 1.705 μm; *N* = 3) and intact side (2.550 ± 1.982 μm; *N* = 3) to the INF/AMP region of mice 5 m following bilateral OVX. The intact side had the largest apical nuclear displacement. (K) Measurements of the distance of SC nuclei to stroma in the INF/AMP region of mice 5 months following unilateral (OVX side = 6.013 ± 2.493 μm, intact side = 5.955 ± 2.642 μm) and bilateral OVX. Each point represents a measurement/cell. The continuous line represents the median, while the dotted lines show the quartiles. ****p < 0.0001, **p < 0.01, *p < 0.05, ^ns^ *p* > 0.05.

To address these possibilities, we performed bilateral ovariectomy (OVX) in 2mo mice and evaluated the short and long‐term impact to oviduct epithelial cells. For short‐term analysis, we isolated the reproductive tracts 1‐month post OVX. In these oviducts, discernible cytoplasmic vacuoles were not observed (Figure 4A), suggesting that MCC vacuolation was not a response to the lack of the estrous cycling. It is of worth to note that the INF/AMP SC nuclei were displaced apically due to SC protrusion from the epithelial monolayer (Figure 4A), similar to the diestrus stage in non‐OVX females.

For the long‐term analyses, we isolated reproductive tracts 5 and 11 months post OVX. At 5 months post OVX, when the age of these mice was 7mo, we observed MCC vacuolation in the INF/AMP epithelium (Figure 4B), showing that the timeline of MCC vacuolation was unaffected by OVX. In agreement with this, vacuoles were not noted in the INF/AMP of mice 3 months post OVX, when the age of the mice was 5mo (Figure S2A). Thus, OVX did not facilitate or prevent MCC vacuolation. Interestingly, the apical displacement of the nucleus in MCCs at 5 months post OVX was not significantly different from 1‐month post OVX (Figure 4D), despite noticeable vacuole formation. Instead, the cellular height of MCCs in mice 5 months post OVX (8.557 ± 1.84 μm) was lower than 1‐month post OVX (9.427 ± 2.244 μm, *p* < 0.01). Taken together, bilateral OVX did not prevent or facilitate MCC vacuolation, but the height of the epithelial cells was decreased. This was reflected in a significant decrease in the apical displacement of INF/AMP SC nuclei in 5 months post OVX despite obvious protrusion from the epithelial monolayer (Figure 4B,E). A large cytoplasmic vacuole was noted in individual INF/AMP MCCs at 11 months post OVX, with an apically displaced nucleus (Figure 4C,D). INF/AMP SC nuclei at 11 months post OVX were also apically displaced relative to 5 months post OVX, due to progressive enlargement of cytoplasmic vacuoles resulting in increased epithelial cell height at 11 months post OVX. Finally, despite cell shape change and loss of estrous cycling/ovulation, there was no significant difference in the proportion of MCCs in the INF/AMP region isolated from mice that were 2‐4mo, or 1 and 11 months post bilateral OVX (Figure 4F), suggesting low epithelial cell turnover, consistent with previous studies (Ford et al. 2023; Roberson et al. 2020). No discernible cytoplasmic vacuoles were noted in the AIJ, ISM, and UTJ at 11 months post OVX (Figure S2B–D).

Since bilateral OVX completely terminates the ovarian hormonal cycle, we performed unilateral ovariectomy surgery in 2mo mice to delineate ovulation and hormonal cycle‐associated homeostatic events in the epithelium. We isolated reproductive tracts 5 months post unilateral OVX (Figure 4G) and found MCC vacuolation on both sides, with no apparent difference between OVX and control sides (Figure 4H,I). Maintenance of estrous cycling without ovulation did not prevent MCC vacuolation. However, apical displacement of MCC nuclei on the OVX side was smaller than the control/intact side. This could be due to lumen and cell shape changes associated with the AMP swelling during/around the time of ovulation (Harwalkar et al. 2021). Additionally, there was no significant difference in MCC nucleus apical displacement between the OVX side and mice 5 months post bilateral OVX (Figure 4J) or in displacement of SC nuclei (Figure 4K). Thus, we conclude that the MCC vacuolation is not caused by repetitive ovulation or estrous cycle‐associated events.

### 2.5 Enrichment of Genesets Associated With ER, Metabolic, Mitochondrial Stress and/or ATP Metabolism in Aged INF/AMP Cells, Suggesting a Metabolically Stressed Microenvironment

**FIGURE 5:**
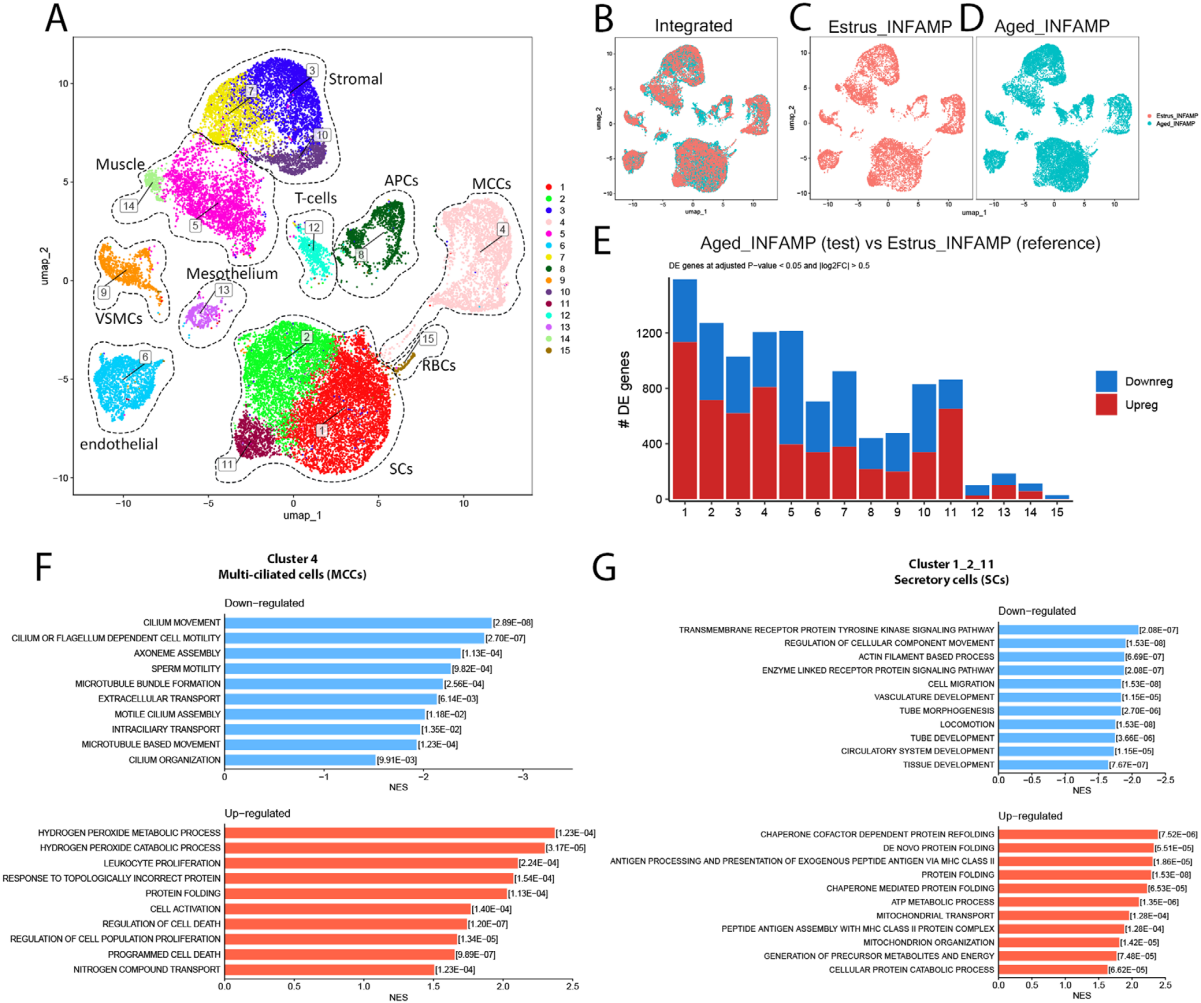
ROS metabolism, ER stress, and cell death regulation gene‐sets were upregulated in 18mo INF/AMP MCCs, while ER stress, ATP metabolism, mitochondrial transport gene sets were upregulated in 18mo INF/AMP SCs. (A) Integrated UMAP plot color‐coded by cluster membership, cell populations are labeled and enclosed within dotted lines, identified based on top 5 genes and known markers (A). (B–D) UMAP clustering of cells isolated from 3mo estrus stage mouse (C), 18mo mice (D), and overlap between the two (B) showing no significant alteration in the isolated populations. (E) Number of differentially expressed genes in each cluster, adjusted for *p*‐value < 0.05 and |log2FC| > 0.5, between INF/AMP cell populations isolated from three 18mo mice (test) and one 3mo estrus stage mouse (reference). (F) Gene sets downregulated (top, blue) and upregulated (bottom, orange) in INF/AMP MCCs of 18mo mice, as compared to a 3mo estrus stage mouse. (F, G) Gene sets downregulated (top, blue) and upregulated (bottom, orange) in INF/AMP SCs of 18mo mice, as compared to a 3mo estrus stage mouse.

To investigate the changes in the aged INF/AMP microenvironment accompanying MCC vacuolation, we performed single‐cell RNASeq of 16,948 cells pooled from the INF/AMP region of three 18mo mice (Aged_INFAMP) and compared this to 10,281 cells isolated from the INF/AMP of a single estrus stage 3mo mouse (Estrus_INFAMP). Vaginal smears from these 18mo mice were consistent with diestrus stage (2/3), or suggestive of a postcyclic anovulatory condition called persistent vaginal cornification (1/3) (Felicio et al. 1984). Cells from the INF/AMP region of the mouse oviduct clustered into 15 clusters that were then grouped into 10 cell types/populations based on a combination of their top 5 highly expressed genes and known cell subtype markers (Figures 5A, S3, and S4A–M). These cell populations include: 1—secretory cells/SCs (cluster 1, 2, 11 expressing *Epcam*, *Wt1*, *Pax8*, *Ovgp1*, *Sox17*; Figures S3 and S4A–D), 2—stromal cells (cluster 3, 7, 10 expressing *Dcn*, *Igfbp6*, *Pdgfra*; Figures S3 and S4F), 3—multi‐ciliated cells/MCCs (cluster 4 expressing *Epcam*, *Wt1*, *Foxj1*; Figures S4A,B,E), 4—smooth muscle cells (cluster 5, 14 expressing *Acta2*, *Actg2*, *Dmd*; Figure S3), 5—endothelial cells (cluster 6 expressing *Pecam1*, *Sox17*; Figure S4D,G), 6—antigen presenting cells/APCs (cluster 8 expressing *Cd52*, *H2‐Aa*; Figures S3 and S4H), 7—vascular smooth muscle cells/VSMCs (cluster 9 expressing *Acta2*, *Rgs5*; Figure S3), 8—T‐cells (cluster 12 expressing *Cd52*, *Cd3e*; Figure S4H,I), 9—mesothelial cells (cluster 13 expressing *Wt1*, *Msln*, *Dcn*, *Igfbp6*; Figures S3 and S4B,H), and 10—red blood cells/RBCs (cluster 15 expressing *Hbb‐bs*, Figure S3). Surprisingly, despite the apparent morphological differences, there was no difference in cell subtypes and populations. All populations in Estrus_INFAMP and Aged_INFAMP overlapped with each other (Figure 5B). In most clusters, cells from Aged_INFAMP had more upregulated genes than downregulated genes, relative to Estrus_INFAMP (Figure 5C).

Using Gene Set Enrichment Analysis (GSEA), we found that aged 18mo INF/AMP MCCs showed downregulation of genes associated with cilia movement and organization, and upregulation of genes associated with hydrogen peroxide metabolism, unfolded protein response (UPR), and negative regulation of cell death (Figure 5F). This reflects an adaptive/compensatory response to cellular stress, in particular, endoplasmic reticulum (ER) stress in MCCs. Further, aged 18mo INF/AMP SCs showed downregulation of genes associated with transmembrane receptor protein tyrosine kinase signaling, actin filament‐based process, and cell migration/movement. Similar to the MCCs, they showed upregulation of genes associated with stress responses such as UPR, ATP metabolic process, and mitochondrial organization/transport (Figures 5G and S5A–C). We also examined the epithelial subset (clusters 1, 2, 4 and 11), but did not note differences in epithelial cell clustering between Estrus_INFAMP and Aged_INFAMP (Figures S6A–E and S7). Similar genesets were up/downregulated in most epithelial clusters (Figure S8) except the *Krt5*+ cluster 12, reported to be a cancer‐prone, transitional preciliated cell state (Flesken‐Nikitin et al. 2024), which showed downregulation of genesets associated with cell cycle regulation (Figure S8L).

Stromal cells were divided into three clusters and showed downregulation of genes associated with ECM production in clusters 3 and 7 (Figure S9A,B), ribosome biogenesis, and RNA processing in cluster 10 (Figure S9C). Cluster 3 stromal cells showed upregulation of gene sets associated with various biosynthetic and metabolic processes, negative regulation of cell death, and immune system activity regulation, reflecting adaptive mechanisms in response to cellular stress and/or a gradual decline in metabolic efficiency (Figure S9A). On the other hand, cluster 7 stromal cells did not show significant upregulation of gene sets, as compared to 3mo cells (Figure S9B). Cluster 10 stromal cells showed upregulation of genes associated with acute inflammatory response, cell adhesion, and cytokine production (Figure S9C), reflecting a response to inflammation/resolution of inflammation, facilitation of tissue repair, and/or cellular senescence.

Cluster 5 smooth muscle cells showed downregulation of genes associated with muscle organization, blood circulation, RNA metabolic processes, and ribosome biogenesis, and upregulation in tissue growth, cell adhesion, and negative regulation of cell death (Figure S9D). There was no enrichment of genesets in cluster 14 smooth muscle cells (Figure S9F). Taken together, most populations were typically responding to cellular metabolic stresses, with a shift/decrease in biogenesis activities and facilitation of tissue repair to maintain structural integrity. VSMCs showed downregulation of genes associated with tissue growth, while upregulation in genesets associated with vascular tone alteration, regulation of IGFR signaling, removal of ROS/RNS species, ATP metabolism, and monocarboxylic and carbohydrate metabolism (Figure S9E).

Among immune cells, T‐cells and Antigen Presenting Cells (APCs: B‐cells, macrophages, dendritic cells) showed upregulation of genesets associated with UPR (Figure S9G,H), suggestive of a response to cellular stress. Endothelial cells showed downregulation in tissue and blood vessel growth and upregulation in antigen presentation, superoxide radical/hydrogen peroxide removal, and various metabolic processes (Figure S9I). Finally, the mesothelial cells that line the INF/AMP serosa showed downregulation in nerve function and ECM organization while upregulation in UPR, mitochondrial transport/organization, ATP metabolism, and regulation of cell death (Figure S9J), suggesting ER stress, energy demand compensation, and response to cellular stress.

Taken together, the single‐cell transcriptomes of all isolated cells from 18mo INF/AMP indicated a general reduction of biogenesis and growth‐related genes, and upregulated stress response genes and ATP energy demand compensation, without a clear indication of inflammation (Figure S9L).

### 2.6 Hypoxia or Hydroxyurea (HU) Treatment Induced MCC Vacuolation In Vitro Organotypic Slice Cultures

It has been shown that cellular vacuolation in in vitro cultures occurs in response to ER stress, ATP depletion/shortage, cellular senescence, metabolic imbalance, bacterial toxins/viral components, exposure to drugs/toxic chemicals/pollutants, and hypoxia (Denoyelle et al. 2006; Henics and Wheatley 1999; Shubin et al. 2016). To determine the potential cause of MCC vacuolation, we developed an in vitro organotypic slice culture system (150 μm thick slices, see Methods). Since many of the upregulated genesets in 18mo MCCs and SCs were associated with ER stress (Figure 5F,G), we treated INF/AMP slice cultures with 5 μM tunicamycin (TUN) for 24 h to induce ER stress. However, TUN treatments did not result in any noticeable cellular changes (Figure S10A,B), suggesting that MCC vacuolation was not a consequence of ER stress.

**FIGURE 6:**
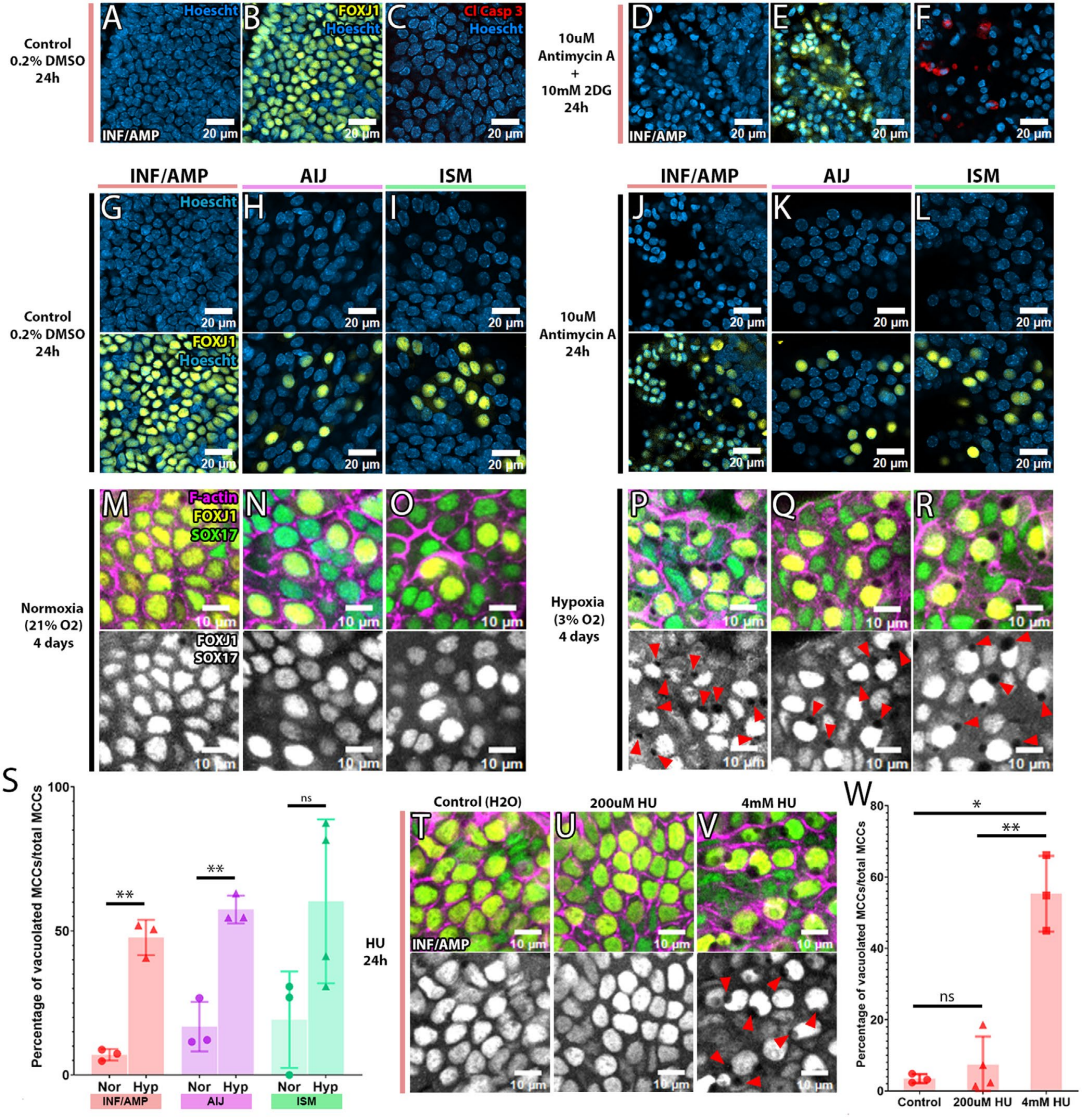
Hypoxia or HU treatment induced MCC vacuolation. (A–F) Inhibition of cellular respiration with 10 mM antimycin A and glycolysis with 10 mM 2DG in organotypic slice cultures of the INF/AMP region. (A–C) Control cultured slice from INF/AMP region with round nuclei (A), of which most cells were FOXJ1+ MCCs (B) and Cleaved Caspase 3‐negative, suggesting no apoptotic cells (C). (D–F) Treatment with antimycin A and 2‐DG for 24 h resulted in MCCs with high intensity nuclear staining (D, E), of which some cells were positive for Cleaved Caspase 3, suggesting apoptotic cells (F). (G–L) Inhibition of cellular respiration in organotypic slice cultures of the INF/AMP (G, J), AIJ (H, K), and ISM (I, L). A 24‐h‐long antimycin A treatment resulted in MCC protrusion from the epithelial monolayer and high intensity of nuclear staining (J) but did not affect AIJ and ISM MCCs (K, L). (M–R) Organotypic slice culture for 4 days in normoxia (M–O) or hypoxia (P–R). When cultured in hypoxia, FOXJ1+ MCCs of the INF/AMP (P), AIJ (Q) and ISM (R) present with empty vacuoles and deformed nucleus, while very few vacuoles formed in normoxia (M–O). FOXJ1 and SOX17, marking oviduct MCCs and SCs, were changed to white for easier visualization of vacuoles (bottom). Red arrows mark the vacuoles. (S) Percentage of vacuolated MCCs among INF/AMP, AIJ, and ISM MCCs in normoxia (7.005% ± 1.995%, 16.971% ± 8.603%, 19.205% ± 16.739% respectively; *N* = 3) versus hypoxia (47.728% ± 6.129%, 57.45% ± 4.821%, 60.283% ± 28.439% respectively; *N* = 3). Increased instances of vacuolation were noted in hypoxia, as compared to normoxia. Error bars indicate standard deviation. Datapoints/replicates are represented by circles (normoxia) or triangles (hypoxia). **p < 0.01. (T–V) Hydroxyurea (HU) treatment for 24 h in INF/AMP organotypic slice cultures. A 200 μM/low dose HU treatment did not result in discernible cellular changes (T, U), while 4 mM/high dose HU treatment resulted in cytoplasmic vacuolation and nucleus deformation (V). FOXJ1 and SOX17, marking oviduct MCCs and SCs, were changed to white for easier visualization of vacuoles (bottom). (W) Percentage of vacuolated MCCs in the INF/AMP when treated with water (2.786% ± 0.573%; *N* = 3), low dose HU (7.397% ± 7.865%; *N* = 3), and high dose HU (55.32% ± 10.622%; *N* = 3) for 24 h. Increased instances of vacuolation were noted in high dose HU treatment, as compared to low dose or water control. Error bars indicate standard deviation. Datapoints/replicates are represented by circles (control), triangles (200 μM HU), or squares (4 mM HU). **p < 0.01, ^ns^ *p* > 0.05, *p < 0.05.

ATP depletion using a combination of antimycin A and 2‐deoxyglucose (2‐DG) treatment results in internalization of Na, K‐ATPase in MDCK cells (Mandel et al. 1994). The combined treatments of these two inhibitors visibly affected INF/AMP MCCs after 24 h. Many MCCs protruded from the epithelial monolayer, along with condensed nuclei and occasional cleaved caspase 3 expression (Figure 6A–F), suggesting apoptotic MCCs. While antimycin A treatment alone also resulted in similar MCC protrusion in INF/AMP MCCs (Figure 6G,J) and termination of cilia beating (data not shown), 2‐DG alone did not (Figure S10C–E), suggesting that INF/AMP MCCs were highly dependent on cellular respiration. Antimycin A‐induced MCC protrusion was unique to INF/AMP slices but not observed in AIJ and ISM slices (Figure 6G–L), suggesting region‐specific cellular respiration dependency. This is consistent with a recent study that showed distinct responses to mitochondrial dysfunction in the lung, dependent on cellular context (Xu et al. 2024).

No significant enrichment in gene sets associated with cellular senescence was noted in the single‐cell transcriptomic analysis (Figure S10F). Although we found the absence of Lamin‐B1, often associated with cellular senescence, in the 18mo oviduct (Figure S10G), we discovered that Lamin‐B1 showed a cyclic expression pattern during the estrous cycle in young females, with high intensity nuclear membrane localization in the estrus stage (Figure S10H) and low intensity in the diestrus stage (Figure S10I). We did not find significant changes in the expression of cellular senescence‐associated pseudo‐DNA damage response genes including cell cycle arrest‐associated P21 and P16, or DNA damage marker phospho‐H2Ax (Pospelova et al. 2009) in 18mo and 2mo mice (Figure S10J–Q). Unexpectedly, phospho‐H2Ax nuclear puncta were predominantly observed in INF/AMP MCCs throughout adulthood and in aging (Figure S10N–Q).

Induction of metabolic stress/nutrient deprivation with 2 μM 6‐AN (pentose phosphate pathway inhibitor; Figure S10R), 1 μM YZ9 (PFKFB3/glycolysis inhibitor; Figure S10S), 0.8 μM ST045849 (hexosamine biosynthesis pathway inhibitor; Figure S10T), 1 μM Shikonin (PKM2/glycolysis inhibitor; Figure S10U), and 10 μg/mL CHX (cycloheximide, protein synthesis inhibitor; Figure S10V) for 24 h in in vitro organotypic slice cultures did not result in significant, discernible cellular changes.

Although there was no significant enrichment of gene sets associated with hypoxia in 18mo INF/AMP (Figure S11A), in vitro INF/AMP tissue slices cultured for 4 days in 3% O_2_ (chronic, mild hypoxia) reproducibly induced a cytoplasmic vacuole and deformed nucleus in MCCs (Figure 6P,S), but similar 4‐day‐long culture in 21% O_2_ (normoxia) did not (Figure 6M). Chronic hypoxia results in reduced ATP demand and rate of mitochondrial oxygen consumption due to ROS elevation (Wheaton and Chandel 2011). Thus, the cytoplasmic vacuolation induced by hypoxia could be a MCC response to ATP shortage and not ATP depletion. Interestingly, hypoxia‐induced MCC vacuolation was not restricted to the INF/AMP epithelium but also observed in AIJ and ISM epithelia (Figure 6M–S). No vacuolated SCs were noted in slice cultures of any of the regions (Figures 6M–R and S11B,C). In vitro experiments suggest that MCCs are more sensitive to hypoxia than SCs, while the cause for unique MCC vacuolation in the INF/AMP epithelium in vivo remains in question.

HU treatment of in vitro organotypic slice cultures with low dose HU, known to induce ROS in cycling cells (Somyajit et al. 2017), did not result in discernible cellular changes (Figure 6T,U,W). We also cultured INF/AMP slices from 8mo oviducts and observed MCCs with condensed, protruding nuclei and/or vacuoles in both control and 200 μM HU treated slices (Figure S11D–F). Since vacuoles were already present in INF/AMP MCCs of 7mo mice (Figure 3A,E,F), we treated AIJ and ISM slices from 8mo mice to test if low dose HU could induce vacuoles in aged tissue. However, these slice cultures did not present with discernible cellular changes in both control and treated conditions (Figure S11G–J). Interestingly, 24‐h long treatment with high dose HU, known to cause dNTP depletion and replication stress in cycling cells (Musiałek and Rybaczek 2021), induced a large cytoplasmic vacuole with a deformed nucleus in MCCs (Figure 6V,W).

### 2.7 MCC Vacuolation Limited to the INF/AMP Epithelium In Vivo Correlated with Aging‐Associated Changes in Blood Flow via the Ovarian Artery

Although our in vitro experiments indicated that all oviduct MCCs were sensitive to hypoxic stress and formed cytoplasmic vacuoles, age‐associated MCC vacuolation was uniquely restricted to the INF/AMP MCCs in vivo. This suggests that, in addition to the distinct cellular property of the INF/AMP epithelium (Ford et al. 2023, 2021; Harwalkar et al. 2021), the local environment within the INF/AMP could be different from the rest of the oviduct. Since metabolic stress‐associated gene sets were upregulated, we investigated blood circulation that supplies the reproductive tract. Three arteries feed the female reproductive organs: ovarian, uterine, and vaginal arteries. The ovarian artery is derived from the abdominal aorta, while the uterine and vaginal arteries are derived from the internal iliac artery (Carretero et al. 2017; Chaudhry and Chaudhry 2023). These patterns are evolutionarily conserved between mice and humans despite anatomical differences in the female reproductive tract.

**FIGURE 7:**
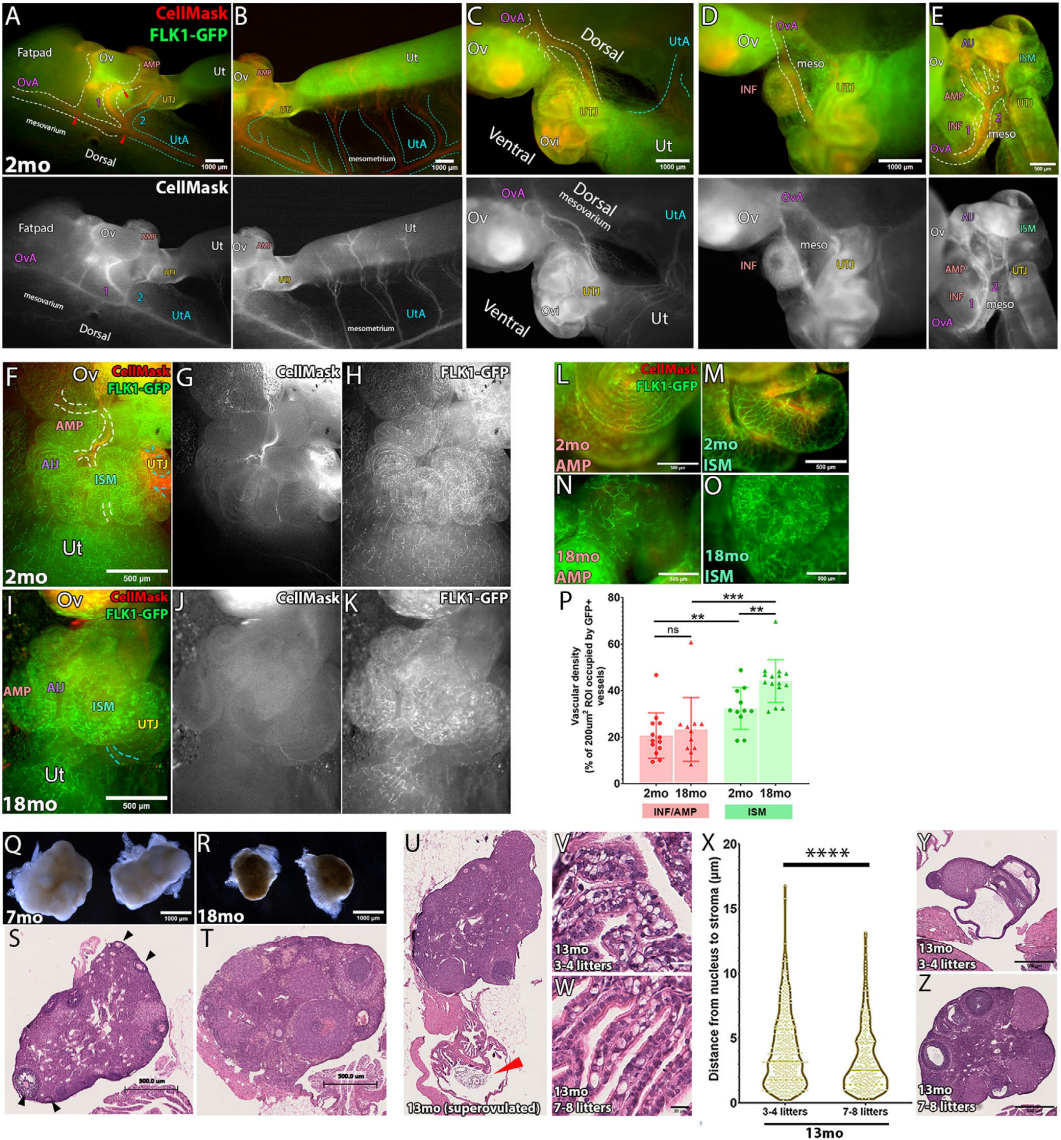
Reduced circulation via the ovarian artery in aged females. (A–E) A 2mo oviduct. Ovarian artery (OvA) and uterine artery (UtA) were labeled by cardiac perfusion of CellMask in a 2mo *Flk1‐gfp* female. The ovarian artery ran along the connective tissue that connected the ovary (Ov), oviduct (Ovi) and uterus (Ut), with branches feeding into the ovary (A). From the anastomosis of the ovarian and uterine arteries, two branches diverged: (1) one fed into the ovary and branched again to supply the distal oviduct (labeled 1, in red), and (2) the other fed into the proximal oviduct (labeled 2, in cyan). The ovarian and uterine arteries were traced by white and cyan dotted lines, respectively. Branching is labeled by red arrows (A). Following the anastomosis point, the uterine artery ran along the broad ligament on the mesometrial side, with numerous branches connecting into the uterus (B). The ovarian artery as seen from a different angle (lateral side facing the observer), with branches that connected into the ovary and the oviduct (C). The oviduct was partially stretched out to visualize a branch of the ovarian artery that ran along the mesosalpinx (D). This artery further branched out: (1) one branch directly fed into the INF/AMP (labeled 1, in red), while (2) the other branch split into numerous vessels that supplied the proximal oviduct (labeled 2, in red) (E). (F–J) Lack of CellMask labeling in in the upper female reproductive tract of aged 18mo mice. Oviducts were oriented such that the ventral coils faced the observer. Little to no CellMask labelling detected in 18mo oviducts (I, J), as compared to 2mo oviducts (F, G) following perfusion. Intricate networks of microvasculature, labeled with FLK1‐GFP, was noted in 2mo (H) and 18mo (K) tracts. (L–P) Microvasculature differences between distal and proximal oviduct. In both young and aged mice, network of FLK1‐GFP expressing vessels was less dense in the AMP (L, N, P), relative to the ISM (M, O, P). No significant differences in vascular density between 2mo AMP (20.70% ± 9.779%; *N* = 3) and 18mo AMP (23.32% ± 13.75%; *N* = 3), but density was higher in 18mo ISM (44.18% ± 9.187%; *N* = 3), relative to 2mo ISM (32.31% ± 9.004%; *N* = 3) (P). Datapoints for 2mo mice are represented by circles, and 18mo mice by triangles. **p < 0.01, ***p < 0.001, ^ns^ *p* > 0.05. (Q–T) Age‐associated ovarian atrophy, decrease in ovary size, and oocyte numbers. Ovaries from 18mo mice were smaller, had a smooth surface (R), and no oocytes were noted (T), as compared to 7mo ovaries (Q, S). (U) MCC function was unaffected by vacuolation. OCCs were noted in the AMP region of 13mo superovulated mice. (V–X) Reduced severity in apical displacement of nucleus in INF/AMP MCCs of 13mo mice with higher gravidity. Fewer vacuolated MCCs were noted in 13mo mice that have had 7–8 litters (W), relative to those that have had 3–4 litters (V). Measurements of the distance from MCC nuclei to stroma in the INF/AMP region of dams with 3–4 litters (3.974 ± 2.713 μm; *N* = 3) or 7–8 litters (3.21 ± 2.411 μm; *N* = 3) (X). Each point represents a measurement/cell. The continuous line represents the median, while the dotted lines show the quartiles. ****p < 0.0001. (Y, Z) 13mo mice that have had 7–8 litters in their lifetime had larger and less atrophic ovaries (Z), as compared to those with 3–4 litters (Y). Meso, mesosalpinx; Ov, ovary; OvA, ovarian artery; Ovi, oviduct; Ut, uterus; UtA, uterine artery.

To track circulation, we performed cardiac perfusion of CellMask, a fixable cell membrane staining fluorescent dye. This allowed us to visualize the blood vessels around the female reproductive tract, particularly the ovarian and uterine arteries (Figure 7A,B). We noticed that a branch of the ovarian artery fed into the ovary and parts of the oviduct (Figure 7C–E), while the uterine artery fed into the proximal oviduct and the uterus (Figure 7A–C). Interestingly, this branch of the ovarian artery ran along the mesosalpinx, branching into two vessels, with one directly feeding into the INF/AMP and the other supplying parts of the proximal oviduct (Figure 7C,D). Since the INF/AMP was supplied by only this branch of the ovarian artery, we speculated that circulation to the ovary was linked to that of the INF/AMP. Further, using the *Flk1‐gfp* mouse line, which labels endothelial cells at embryonic stages (Yamaguchi et al. 1993), we found an intricate network of vessels throughout the upper female reproductive tract, likely marking the microvasculature (Figures 7F,H and S12A–D). FLK1‐GFP+ vessel/vascular density was lower in the AMP, as compared to the ISM (Figure 7L–P), suggesting distal‐proximal differences in microvasculature. While FLK1‐GFP+ vascular density in the ISM of aged mice was higher than in young mice, no significant differences were noted in the density of the AMP between young and aged (Figure 7P). CellMask entered the oviduct in young mice following cardiac perfusion (Figure 7A–G), indicating dilated vessels/active circulation. Interestingly, CellMask was not noted in the oviducts or ovaries of aged 18mo mice (Figures 7I,J and S12E,G), suggesting changes/decline in circulation. Circulation into the ovary declined in tandem with ovarian aging, correlating with a reduction in ovary size and the number of oocytes in aged mice (Figure 7Q–T). Ovarian aging and oocyte depletion progressively occurs in 3–12mo mice, with a decrease in the number of oocytes at 6mo, and pronounced reduction at 9mo and 12mo (Ansere et al. 2021). This timeline of oocyte depletion and ovarian aging occurs concurrently with INF/AMP MCC vacuolation, likely due to changes in circulation via the ovarian artery.

Since aging‐associated decline in female fertility is caused by ovarian aging/oocyte depletion, we wondered if MCC vacuolation contributed to this decline in fertility by hindering oocyte cumulus complex (OCC) movement into the oviduct. Regardless of cytoplasmic vacuoles in MCCs, superovulation of 13mo mice resulted in the successful entry of OCCs into the oviduct (Figure 7U), indicating that vacuolation did not affect INF/AMP functions mediated by MCC beating (Harwalkar and Yamanaka 2021). Indeed, cilia beat frequency (CBF) of INF/AMP MCCs in 13mo mice (25.491 ± 3.76 Hz; Video S1) was not significantly different from 2mo mice (27.27 ± 3.39 Hz, *p* = 0.41; Video S2), suggesting continued ATP consumption of MCCs in aged oviducts.

Pregnancy modulates blood circulation around the female reproductive tract to support fetal growth. It is shown that the uterine artery contributes 68%, and the uterine branch of the ovarian artery contributes 32% to the placenta/fetuses (Raz et al. 2012), indicating that the ovarian artery also supports the continuation of pregnancy. We wondered whether gravidity, defined as the number of pregnancies, would impact INF/AMP MCC vacuolation. Interestingly, INF/AMP MCCs in 13mo mice that have given birth to 7–8 litters in their lifetime (last litter at age 8–9mo/4–5 months prior to euthanasia) presented with decreased severity of vacuolation and apical displacement of the nucleus, relative to 13mo mice that have given birth to 3–4 litters in their lifetime (last litter at age 5–6mo/7–8 months prior to euthanasia; Figure 7V–X). We also found that 13mo mice with 7–8 litters had larger and less atrophic ovaries, as compared to mice with 3–4 litters (Figure 7Y,Z). As expected, MCC vacuolation was restricted to the INF/AMP epithelium and not noted in the other regions of the oviduct (Figure S12H–J). Taken together, we propose that changes in blood circulation via the ovarian artery contribute to ovarian and distal oviduct aging. Reduced blood circulation results in a constant, mildly stressed microenvironment within the INF/AMP region leading to MCC vacuolation.

## 3 Discussion

The recent recognition of the distal end of the fallopian tube as the tissue of origin of high‐grade serous tubal ovarian carcinomas (HGSCs), and that its patient demographic is strongly biased to postmenopausal women, motivated us to investigate age‐associated changes in this organ. In this study, we show that the INF/AMP MCCs uniquely present with aging‐associated cytoplasmic vacuolation and nuclear anomaly. We initially suspected that repetitive exposure to inflammatory factors or ROS released during ovulation (Murdoch and Martinchick 2004) could be part of the cause. However, ovariectomy did not affect the timeline of MCC vacuolation in the INF/AMP epithelium. Additionally, the MCC vacuolation phenotype was restricted to the INF/AMP epithelium and not observed in the AIJ epithelium despite the same proximity to the ovary in a single transverse section. We concluded that MCC vacuolation in the INF/AMP epithelium was not associated with proximity to the ovary but was due to a distinct cellular subtype and microenvironmental changes, suggesting intrinsic vulnerability.

MCCs in the INF/AMP epithelium are constantly beating regardless of the female hormonal cycles and ovulation. Although changes in cilia beat frequencies (CBF) are reported during the different hormonal stages, they do not impact OCC transport along the AMP (Shi et al. 2011). We observed that MCCs in the aged INF/AMP continued to beat and transport OCCs into the AMP despite MCC vacuolation. In in vitro organotypic slice cultures, inhibition of cellular respiration terminated cilia beating (data not shown) and resulted in INF/AMP MCC protrusion/death within 24 h of treatment, with no noticeable changes in SCs or AIJ/ISM MCCs. This is consistent with a recent study showing that lung MCCs and their progenitors respond differently to mitochondrial dysfunction (Xu et al. 2024). Taken together, this suggests that INF/AMP MCCs rely heavily on oxidative phosphorylation to fulfill the high ATP usage required for constant cilia beating.

The most ATP‐consuming biochemical events in adult cells are (1) Na+, K+ ATPase activity responsible for the establishment and maintenance of ion homeostasis, membrane potential, active transport of substrates and (2) protein turnover (Kelly and McBride 1990; Wheaton and Chandel 2011). However, constant MCC beating in the INF/AMP epithelium continued even after the cessation of ovarian functions and MCC vacuolation, suggesting that these cells prioritize cilia function/motility. Acute ATP depletion induced by combined inhibition of glycolysis and cellular respiration in MDCK cells results in the internalization of Na+, K+ ATPase and E‐cadherin (Mandel et al. 1994). Thus, the cytoplasmic vacuoles lined with Na+, K+ ATPase α1 subunit (ATP1A1) observed in INF/AMP MCCs of aged mice could be indicative of cellular adaptation to ATP shortage.

MCC vacuolation was induced in hypoxia (3% O_2_) or high dose HU treatment in in vitro slice cultures, but not by ER stress inducers or inhibition of protein synthesis, glycolysis, hexosamine biosynthesis or pentose phosphate pathway. Acute hypoxia promotes Na+, K+ ATPase internalization by generating mitochondrial ROS (Gusarova et al. 2011), while chronic hypoxia results in ROS elevation and diminished ATP demand (Wheaton and Chandel 2011). In line with this, INF/AMP MCCs in 18mo mice showed downregulation of gene sets associated with cilia function/motility, reflecting decreased ATP demand, and upregulation of gene sets associated with ROS metabolism/catabolism, suggesting that the MCCs are adapting to environmental stress.

HU treatment, known to inhibit DNA synthesis, resulted in MCC vacuolation. HU predominantly affects the S‐phase of the cell cycle, inducing dNTP depletion and replication fork stalling in cycling cells (Musiałek and Rybaczek 2021) by inhibiting ribonucleotide reductase (RNR). Hypoxia also induces dNTP depletion and replication stress in cycling cells (Foskolou et al. 2017), as oxygen is an essential co‐factor for RNRs (Huang et al. 2014). Based on our finding of phospho‐H2AX puncta in INF/AMP MCCs throughout adulthood and aging, it is interesting to speculate that vacuolation may be a response to replicative stress, despite MCCs being terminally differentiated, non‐cycling cells. Interestingly, prolonged HU treatment results in formation of heterogeneous numbers of centrosomes in cycling Chinese Hamster Ovary (CHO) cells, suggesting that HU may countermand the suppression of centriole/centrosome formation (Kuriyama et al. 2007). Thus, in non‐dividing MCCs with numerous centrioles, another possibility is that prolonged, high‐dose HU treatment deregulates centriole numbers.

Although MCCs from all three regions of the oviduct were sensitive to hypoxia and presented vacuolation phenotypes in vitro, the MCC vacuolation phenotype in vivo was restricted to the INF/AMP epithelium. This suggests not only intrinsic vulnerability but also unique microenvironmental differences in the INF/AMP epithelium.

Three arteries feed into the female reproductive tract: ovarian, uterine, and cervical arteries (Chaudhry and Chaudhry 2023). We found that the ovarian artery feeds into the ovary and INF/AMP region, while the uterine artery supports the proximal oviduct. Interestingly, in some Mayer‐Rokitansky‐Küster‐Hauser (MRKH) syndrome patients, the distal end of the Fallopian tube is intact despite the lack of the uterus (Garrett et al. 2008), suggesting that its circulation is independent of the uterus. Branches of the ovarian artery connect into the distal region, while the uterine artery feeds into the proximal Fallopian tube (Verco 1994). Thus, blood circulation patterns in mice and humans are conserved despite apparent anatomical differences between mouse and human female reproductive tracts.

Our data showed blood circulation to the INF/AMP region relied on the ovarian artery, which was tightly linked to ovarian function. In aging, we found reduced circulation to the ovary and the INF/AMP. We propose that this creates a chronic, mild low oxygen and low nutrient condition in the INF/AMP region, reflected by MCC vacuolation.

Finally, the distal fimbria of the human Fallopian tube is recognized as the site of origin of HGSCs (Piek et al. 2001). It is interesting to speculate that low blood circulation to the distal Fallopian tube via the ovarian artery in postmenopausal individuals could lead to a constant, mild environmental stress that facilitates the initiation and progression of HGSCs. In one human study, while variations exist between individuals and hormonal conditions, changes in the ovarian/uterine artery calibers are observed in FTs of cycling and postmenopausal women, suggesting that FTs from cycling humans receive blood supply mainly from the ovarian artery and FTs from postmenopausal humans predominantly receive blood supply from the uterine artery (Brökelmann 1989). In addition, while region‐specificity is unclear, stretches of vacuolated epithelial cells are reported in 6.6% of isolated human FT samples, frequently in older patients of average age 61 years (Hunt and Lynn 2002). We also showed that parity was directly proportional to decreased severity of MCC nucleus displacement and vacuolation, suggesting that pregnancy provided a protective effect. Likewise, the *P53* signature, considered a latent HGSC precursor, is significantly associated with lower parity (Saleemuddin et al. 2008), and thus with an increased number of ovulations. While cytoplasmic vacuolation occurred independent of estrous cycling and/or ovulation, the severity of vacuolation decreased with higher gravidity/parity. Pregnancy involves increased blood flow and oxygenation of the reproductive tract (Raz et al. 2012), including the distal oviduct and ovary, which also prevents shrinkage and decreased atrophy of ovaries in mice with higher gravidity.

Taken together, we propose that the INF/AMP epithelium has unique intrinsic properties and circulatory environment, as compared to the rest of the oviduct. Changes in blood circulation were associated with cessation of ovarian function and resulted in environmental stress that was reflected in INF/AMP‐specific MCC vacuolation.

## 4 Materials and Methods

### 4.1 Sex as a Biological Variable

Our study exclusively examined female mice because the organs studied are only relevant in females.

### 4.2 Animals

Our study exclusively examined female mice because the organ is only relevant in females. All animal work was performed in accordance with institutional guidelines and was approved by the Faculty of Medicine Animal Care Committee (AUP #7843); it was undertaken at the Goodman Cancer Research Institute animal facility. Aging phenotypes were confirmed in *Fltp‐H2B‐Venus* (Gegg et al. 2014) mice that were a kind gift from Dr. Heiko Lickert and Tdtomato flox/flox mice (Ai14) acquired from JAX (#007914). The *Flk1‐gfp* mouse line was a kind gift from Dr. Janet Rossant. Wild‐type B6N mice acquired from McGill's Integrated Core of Animal Modeling (MICAM) were used for ovariectomy surgeries (*N* = 3–4 for each time‐point), and results were confirmed in *Fltp‐H2B‐Venus* mice (*N* = 2–3 for each time‐point). A 13mo retired B6N dams with 3–4 (*N* = 3) or 7–8 litters (*N* = 3) were acquired from MICAM, where trio breeding is the standard. Where required, estrous stages were analyzed using vaginal smears stained with crystal violet (McLean et al. 2012).

### 4.3 3D Confocal Imaging

Oviducts were collected and straightened by removing the mesosalpinx. The straightened oviduct was fixed with DMSO (Dimethyl sulfoxide, Sigma Aldrich D8418): methanol (BioShop Canada Inc., MET302.1) in the ratio 1:4 and placed at −20°C overnight. The antibody staining protocol was as described in (Arora et al. 2016). Blocking was done overnight in a solution containing 1% Triton X‐100 (Sigma‐Aldrich, T8787), 2% powdered milk, and 1× phosphate‐buffered saline (PBS, BioShop Canada Inc., PBS405), henceforth referred to as PBSMT. Primary and secondary antibody staining was performed in PBSMT for 5 and 2 days, respectively, at 4° on a shaker. After six 30‐min‐long PBSMT washes, oviducts were transferred successively to methanol:PBT (1:1, PBT: 1× PBS and 1% Triton X‐100), 100% methanol, 3% H_2_O_2_ (BioShop Canada Inc., HYP001.1), and 100% methanol prior to benzyl alcohol/benzyl benzoate (BABB) clearing. BABB‐cleared samples were placed on a #1.5 coverslip (Fisher Scientific, 12‐545F) with 10–15 μL BABB prior to imaging using the 10× objective (0.30 numerical aperture/N.A) on LSM 800 or 710 (Zeiss). Section interval for 3D confocal imaging was 4.32 μm. Usually, 40–110 optical sections were taken.

### 4.4 Bilateral and Unilateral Ovariectomy Surgeries

Surgery preparation and female reproductive tract exposure was as described previously (Harwalkar et al. 2023). Mice were anesthetised with isoflurane (McGill's Comparative Medicine and Animal Resources Centre/CMARC pharmacy) and injected with 20 mg/kg subcutaneous Carprofen (CMARC pharmacy) prior to fur removal and surgery. A dorsal midline skin incision (~1 cm) was created, followed by an incision in the body wall located directly above the ovarian fat pad. This ovarian fat pad was pulled out using a pair of curved forceps (Fisher Scientific, NC0696845), exposing the upper female reproductive tract (ovary, oviduct, uterus). The tract was placed on a sterile gauze soaked in saline and a bulldog clamp (Fisher Scientific, 50‐822‐230) was secured on the fat pad to keep the tract exposed. Under the microscope (SteREO Discovery.V8, Zeiss), the ovarian bursa was cut open to expose and cut out the ovary using a pair of sterile microdissection scissors (Fisher Scientific, 089531B). Following ovary removal, body wall and skin incisions were sutured closed using absorbable vicryl sutures (Fisher Scientific, 50‐118‐0847). Mice were administered Carprofen for 3 days and monitored for 10 days.

### 4.5 Immunofluorescence and Immunohistochemistry

Uncoiled or coiled oviducts/reproductive tracts were fixed with 4% paraformaldehyde (PFA, Polysciences) for 30 min, followed by three washes with 1× PBS. For cryosectioning, fixed samples were calibrated in 15% and 30% sucrose either at 4° overnight prior to OCT (Fisher HealthCare, #23‐730‐571) embedding and sectioning at 5–7‐ or 10‐μm thickness. For immunofluorescence (IF), sections were brought to room temperature and washed with 1× PBS, 0.1% Triton X‐100, and 0.5% Triton X‐100. Slides were incubated in permeabilization and blocking solution (0.1% Triton X‐100 and 1% FBS in 1× PBS) for 2 h at room temperature. Primary antibody staining was performed overnight at 4°C in permeabilization and blocking solution. Primary antibodies were washed off with multiple washes of 1× PBS and 0.1% Tween (Sigma Aldrich, P9416), followed by secondary antibody staining in 1× PBS overnight at 4°C. After the secondary antibodies were washed off, one to two drops of homemade mounting media were pipetted onto the sections, and #1.5 coverslips were placed over the stained sections. Coverslips were sealed to the slides using nail polish. IF sections were imaged with 20× (N.A. 0.8) or 63× (N.A. 1.4) objectives, on LSM710 or LSM800 confocal microscopes (Zeiss). Homemade mounting media was made of 1 part 10× PBS, 9 parts glycerol (BioShop, GLY001), and slowly stirred in 0.1 parts of 20% w/v propyl gallate (Sigma Aldrich, P3130; dissolved in DMSO). Paraffin processing, hematoxylin & eosin (H&E), immunohistochemistry (IHC), and digital slide scanning (NanoZoomer S210, Hamamatsu) were performed by the Goodman Cancer Research Institute Histology Innovation Platform. Embedded blocks were sectioned on the microtome at 4–6‐μm thickness. For IF on paraffin sections, deparaffinization involved heating at 50°C, two incubations of 5 min each in Histo Clear (National Diagnostics, HS‐202) and 3‐min‐long incubations in successive ethanol solutions (100%, 90%, 70%, 50%, 0% in water). Antigen retrieval was performed with homemade 1× buffer (20 mM Tris, 1 mM EDTA, 0.05% Tween‐20 at pH 9.0) for 7 min in a pressure cooker. Primary and secondary antibody staining was performed as stated above.

The 150 μm thick organotypic slice cultures were transferred to four‐well plates and fixed in 4% PFA for 30 min at room temperature, on a shaker. PFA was replaced by 1× PBS thrice, each wash with shaking for 10 min at room temperature, and 1× PBS was replaced by permeabilization and blocking solution and incubated overnight at 4°C on a shaker. Primary antibody staining was performed in permeabilization and blocking solution overnight at 4°C on a shaker, followed by multiple 1erformed with homema PBS and 0.1% Tween washes, secondary antibody staining in 1× PBS on day 3, and multiple washes. Slices were transferred to a #1.5 coverslip with a 120 μm spacer stuck on the surface. Any solution remaining on the coverslip was replaced by a drop of homemade mounting media, followed by a second #1.5 coverslip on top, such that the slices were sandwiched between two coverslips. Stained slices were imaged with a 20× objective (0.8 N.A) on LSM710 or LSM800 confocal microscopes.

### 4.6 Antibodies

Primary antibodies (1/250 dilution, unless specified): anti‐SOX17 (R&D Systems, AF1924), anti‐PAX8 (Proteintech, 10336‐1‐AP), anti‐FOXJ1 (Abcam, ab235445), anti‐E‐cadherin (Sigma Aldrich, U3254), anti‐GFP (Abcam, ab13970), anti‐ATP1A1 (1/20 dilution, a2F, DSHB), anti‐LaminB1 (Cell Signaling, 13435S), anti‐Cleaved Caspase 3 (Cell Signaling, 9664S), anti‐ᵞH2AX (Cell Signalling, 9718T), anti‐P16 (Cell Signalling, 92803T), anti‐P21 (Cell Signalling, 2947T).

Secondary antibodies (1/450 dilution): Alexa Fluor (AF) anti‐rabbit 555 (Invitrogen, A31572), AF anti‐rabbit 649 (Invitrogen, A21245), AF anti‐rabbit 488 (Invitrogen, A21206), AF anti‐mouse 649 (Invitrogen, A32787), AF anti‐mouse 488 (Invitrogen, A21202), AF anti‐goat 568 (Invitrogen, A11057), AF anti‐rat 488 (Invitrogen, A21208), anti‐chicken 488 (Sigma, SAB4600031), DAPI (4′,6‐diamidino‐2‐phenylindole, Thermo Fisher, 62248), Hoescht 33342 (Thermo Fisher, 62249), AF 488 phalloidin (Lifetech, A12379), and AF 635 phalloidin (Lifetech, A34054).

### 4.7 Isolation of INF/AMP Cells for Single Cell RNA Sequencing

Oviducts from a single 3mo mouse in estrus (Estrus_INFAMP) or three 18mo mice (Aged_INFAMP) were straightened out in 1× PBS. The INF/AMP region was separated from the rest of the tube by slicing in between turns 1 and 2. INF/AMP regions were moved into media containing 1× PBS, 100 IU/mL of penicillin and 100 ug/mL of streptomycin (Gibco, 15140122), and 10% fetal bovine serum (FBS, Wisent Bioproducts, 090‐150). These pieces of tissue were transferred onto empty plates with a drop of media containing 5 mg/mL collagenase B (Sigma‐Aldrich, COLLB‐RO) and 5 U/100 μL DNase I (Thermo Scientific, EN0521) and chopped into smaller pieces with a No. 21 blade (Paragon, P307). This solution with small tissue pieces was transferred to a 1.5 mL tube containing 100 μL of the above‐mentioned dissociation solution and incubated for 35–40 min at 37°C. This was then spun down at 1500 rpm for 5 min, resuspended in 500 μL 1× PBS, and spun down again to resuspend in 50 μL of warmed 0.25% Trypsin–EDTA (Gibco, Cat# 25200056) followed by a 7‐min incubation at 37°C. Trypsinization was halted with media, and mechanical dissociation was performed by passing through a 27 ½ gauge insulin needle (BD, #324704). The resulting single‐cell suspension was passed through a 40 μm cell strainer (Corning, 352340), centrifuged at 1500 rpm for 7 min, and resuspended in 200 μL 1× PBS.

### 4.8 Single Cell RNA Sequencing (scRNASeq)

Quality control and scRNASeq was performed by the McGill Genome Centre. For initial quality control, an aliquot was taken from the cell suspension and incubated in a working concentration of 2 mM calcein‐AM and 4 mM Ethidium‐Homodimer1 (Thermofisher L3224). After 10 min of incubation at room temperature, sample viability, concentration, segregation, size, and absence of large debris were verified by loading the stained cell suspension onto a hemocytometer (Incyto DHC‐N01‐5) and imaging on bright field, GFP (for Calcein‐AM) and RFP (for Ethidium homodimer‐1) channels using an EVOS FL Auto Fluorescent microscope (ThermoFisher). Samples with a viability of 70% or more proceeded to scRNASeq. Single cell gene expression data was generated according to the Chromium Single Cell User Guide (v3.1 Chemistry, 10× genomics). Briefly, cells were suspended into Reverse Transcription (RT) Master Mix (10× genomics) then pipetted into Well‐1 of a Chip “G” (10× genomics), followed by Gel Beads (10× genomics) and Partition oil (10× genomics). The chip assembly was run on a Chromium Controller (10× genomics) which generated Gel Bead‐In‐EMulsions (GEMs). Following the RT protocol on a thermocycler (Biorad T100), emulsion beads were broken, and the quality (size distribution) and concentration of cDNA were assessed using LabChip (Perkin Elmer 760517, CLS760672). Barcoded cDNAs were pooled for amplification, and the final PCR product (or sequence ready library) was purified and quality controlled using LabChip. Finally, the libraries were sequenced on 1 lane of MGI G400, DNBSEQ‐G400 sequencer (MGI Tech Co. Ltd).

### 4.9 scRNASeq Analyses

Droplet libraries were processed using the Cell Ranger count pipeline (v7.0.1) (Zheng et al. 2017). Sequencing reads were aligned to the mm10 mouse reference genome, and transcript counts were quantified for each annotated gene within every cell. Count matrices (genes × cells) were loaded into the R package Seurat (v5.0.1) (Hao et al. 2021) for quality control (Figure S13A–D) and downstream analyses. Low‐quality cells were filtered out using the following criteria: (1) the number of detected genes is ≤ 200; (2) percentage of mitochondrial RNA ≥ 10% per cell. Following SCTransform normalization, individual samples were integrated using the Harmony procedure. UMAP dimension reduction was generated based on the first 15 principal components. A nearest‐neighbor graph using the first 15 principal components was calculated using the FindNeighbors function, followed by clustering using the FindClusters function with resolution = 0.35. Cluster‐specific marker genes were identified using the function FindMarkers with cutoffs: log2 fold change > 0.5 and adjusted *p*‐value < 0.05 (up‐regulated genes only). Per‐cluster differential expression testing between two groups of cells was conducted using the FindMarkers function. The Wilcoxon rank‐sum test was utilized for the differential analysis, and *p*‐values were corrected for multiple testing using the Benjamini–Hochberg method. Gene set enrichment analysis based on a pre‐ranked gene list by *t*‐statistic was performed using the R package fgsea (http://bioconductor.org/packages/fgsea/). To assign epithelial cells a detailed type annotation, we subjected the epithelial clusters to an additional round of subclustering as described above with resolution = 0.6.

### 4.10 Live Imaging Cilia Beating

INF regions of the oviduct were cut off and sliced radially in warm 1× PBS. These pieces were gently transferred to a #1.5 coverslip (VWR, 48393‐251) with a 120 μm spacer (Invitrogen, S24737) stuck on the surface, and any remaining PBS was replaced with ~10 μL of warmed media containing Advanced DMEM/F12 (Gibco, 12634010), 2 mM GlutaMax (Gibco, #35050061), 5% FBS, and 50 U/mL penicillin/streptomycin. A second #1.5 coverslip was placed on top to sandwich the tissue pieces, and they were imaged within 30 min using a 20× objective (0.75 N.A) on a widefield microscope system (Nikon), built around a Nikon TI2‐E stand, equipped with Nikon's Perfect Focus System, a LED light source (SpectraX, Lumencor) and sCMOS cameras (Orca Fusion‐BT, Hamamatsu) enclosed by a custom‐built environmental chamber (Digital Pixel), and controlled using Nikon NIS Elements AR software. 2000 loops of 512 × 512 ROIs/tissue edges covered with MCCs were imaged at 37°C. Framerate was calculated for each video by using timestamps from the metadata using the following: number of frames/(last timestamp‐first timestamp); the average framerate was ~400 fps. Cilia Beat Frequency (CBF) was calculated manually using the following: CBF (Hertz) = (frame rate/number of frames taken for 10 beat cycles to occur) × 10.

### 4.11 Superovulation

13mo mice were administered 100 μL of CARD HyperOva (Cosmo Bio Ltd., KYD‐010‐EX) intraperitoneally at 5 pm. Forty‐eight hours later, 7.5 IU of human Chorionic Gonadotropin (hCG; Sigma, CG‐10) was administered intraperitoneally. Mice were euthanized 15–17 h after hCG injection, and tracts were isolated for paraffin processing.

### 4.12 Organotypic Slice Culture

Reproductive tracts were dissected out of 2‐3mo or 8mo mice and dropped into a petri dish containing ice‐cold 1× PBS to reduce contractions. Ovaries were removed, and oviducts were straightened out by removing the mesosalpinx. ~1 mL of warmed 4% low melting agarose (LMA, IBI Scientific, IB70051) was pipetted into a cryomold (Fisherbrand, 22363553). The straightened oviduct was embedded in the LMA and placed on ice to set. A portion of the uterus was kept intact to stabilize the tract in the LMA while sectioning. The solidified LMA block was superglued (Krazy Glue) onto the metal specimen holder such that the cut side of the uterus faced the specimen holder, and the distal tip of the uncoiled oviduct faced the ceiling. After the glue dried, the specimen holder was placed in the buffer tray containing ice‐cold 1× PBS, surrounded by ice in the ice bath tray. The block was sectioned into 150 μm thick, transverse slices at a speed and amplitude of 0.8 mm/s and 1.60 mm, respectively, using a vibratome (Leica). Slices were picked up using a brush and placed in a well of a 24‐well plate (Fisher Scientific, FB012929) containing 250 μL of ice‐cold media. Tissue slices embedded in agarose were transferred to a 4‐ (Fisher Scientific, Cat# FB012926) or 25cm^2^ dish (Fisher Scientific, 08‐757‐500) and 500 μL/8 mL of fresh, ice‐cold media was added to each well/dish. Media contained Advanced DMEM/F12, 2 mM GlutaMax, 5% FBS, and 50 U/mL penicillin/streptomycin.

25 cm^2^ dishes were used for experiments comparing normoxia to 3% hypoxia, while 4‐well or 24‐well plates were used for all other treatments. Organotypic slice cultures in 25cm^2^ dishes were placed in normoxia or hypoxia on the day of sectioning. Hypoxia (3.5% O_2_, 5% CO_2_, 91.5% N_2_) was maintained in a separate, humidified CO_2_ incubator with O_2_ control (Heracell 150i, ThermoFisher). Organotypic slice cultures in 4‐well plates were placed in a humidified CO_2_ incubator for 24 h prior to inhibitor treatments. Inhibitors/treatments used were 200 μM/4 mM hydroxyurea (HU, Sigma Aldrich, H8627‐5G), 10 μg/mL cycloheximide (CHX, Cayman Chemical Company, 14126), 2 μM 6‐Aminonicotinamide (6‐AN, Sigma, A68203‐1G), 1 μM YZ‐9 (Cayman Chemical Company, 15352), 5 μM tunicamycin (TUN, Sigma Aldrich, T7765‐10MG), 1 μM shikonin (Cayman Chemical Company, 14751), 0.8 μM ST045849 (Sigma Aldrich, SML2702‐5MG), 10 μM antimycin‐A (Sigma Aldrich, A8674), and/or 10 mM 2‐deoxyglucose (2‐DG, Sigma Aldrich, D6134).

### 4.13 Cardiac Perfusion for Blood Vessel Visualization

Mice were anesthetized with isoflurane, and the cardiac chamber was opened to expose the heart. A catheter was inserted into the left ventricle, the right ventricle was cut open, and 30 mL of 0.85% saline was injected to flush the arteries. After flushing, 4 mL of 0.25% CellMask (Invitrogen, C10045), diluted in 0.85% sterile saline, was injected into the left ventricle. The female reproductive tract was dissected and imaged under a stereomicroscope (SteREO Lumar. V12, Zeiss).

### 4.14 Image Analysis and Statistics

Stained sections were visualized using FIJI, Zen (Zeiss) or ImageScope ×64 (Aperio). Apical displacement of nuclei was measured manually on FIJI by drawing a line from the base of the nucleus to the basal lamina/stroma as parallel to the lateral domain as possible, using either 63× IF images taken on a confocal microscope or 40× H&E‐stained digital slides that were converted to TIFF format using ndpitools and ndpi2tiff plugins. Cellular/epithelial monolayer height was measured manually on FIJI by drawing a line from the base to the apical side of MCCs. Measurements were performed from base to base for each mucosal fold to include cells at the base, side, and tip of each mucosal fold. Apical displacement and cell height measurements were compared between mice of the same genotype and sample preparation.

Cell counts were performed on 20× confocal images using the Cell Counter plugin. Although FLTP‐H2B‐Venus and FOXJ1 were used interchangeably as MCC markers, cell counts were compared only between samples stained with the same marker. The number of MCCs in organotypic slice cultures was first counted on a single z‐stack, and then vacuolated MCCs were identified by inspecting the neighboring stacks. To measure FLK1‐GFP+ vascular density, epifluorescence images were converted into a binary image. 200 μm^2^ regions of interest (ROI) were selected and the area fraction was measured on FIJI. GraphPad Prism was used to perform unpaired, two‐tailed *t*‐test or one‐way ANOVA, and create graphs/plots.

## Author Contributions

K.H. conducted most experiments, acquired data, and analyzed experiments. N.Y. performed the ovariectomy surgeries and provided aged mice with multiple litters. A.S.P. performed bioinformatic analyses. S.Z., A.F.T., V.L., M.T., K.T., and M.J.F. performed some sectioning and/or staining. Y.Y., K.H., and W.P. performed the blood vessel visualization experiments. K.H., W.P., M.J.F., and Y.Y. edited the manuscript. K.H. and Y.Y. designed experiments and wrote the manuscript.

## Conflicts of Interest

The authors declare no conflicts of interest.

## Supporting information


Video S1.

Video S2.

Figure S1.

## Data Availability

The data that supports the findings of this study are available in the Videos S1 and S2; Figures S1–S13 of this article.
