# Supplementary material for: Aging‐Associated Vacuolation of Multi‐Ciliated Cells in the Distal Mouse Oviduct Reflects Unique Cell Identity and Luminal Microenvironment
Source: Aging Cell. 2025 May 1;24(7):e70051. doi: 10.1111/acel.70051 (PMC12266777; doi:10.1111/acel.70051)
Supplement: Supplementary file 3 — Figure S1. [file ACEL-24-e70051-s001.pdf]

SUPPLEMENTARY FIGURES

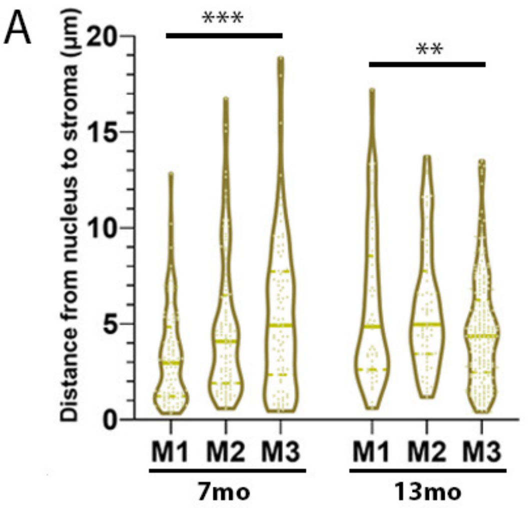

Age	Biological replicate	Mean±SD µm
7mo	M1	3.252±2.465
	M2	4.835±3.697
	M3	5.432±4.012
13mo	M1	5.668±3.877
	M2	5.793±3.129
	M3	4.624±2.806

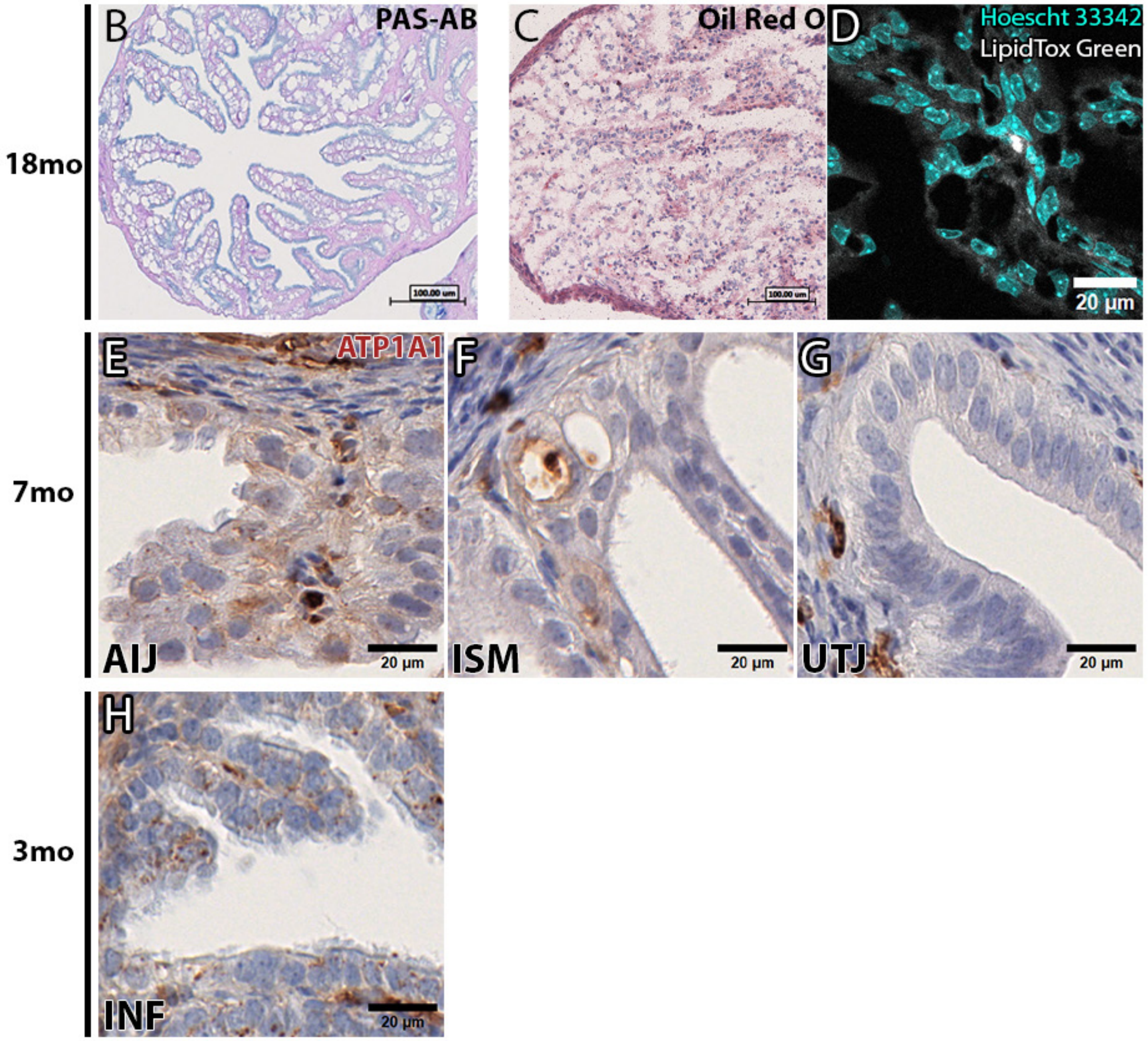

Suppl. Fig. 1: Aging-associated cytoplasmic vacuoles in INF/AMP MCCs do not contain glycogen or lipids, and no ATP1A1 localization was noted in the proximal oviduct. (A) Apical displacement of INF/AMP MCC nucleus is variable between biological replicates, particularly in 7mo mice. Measurements of the distance between MCC nucleus to stroma in individual mice corresponding to Fig. 3C, visualized by violin plots (left), are shown in the table (right). The continuous line represents the median, while the dotted lines show the quartiles. One oviduct per mouse/biological replicate was used for quantification. M1 = mouse 1, M2 = mouse 2, M3 = mouse 3. One-way ANOVA was performed to gauge significance. *** = $p < 0.001$, ** = $p < 0.01$. (B) Periodic acid-Schiff/PAS staining of the 18mo INF/AMP region (N=3). Cytoplasmic vacuoles were negative for PAS, indicating that vacuoles do not contain polysaccharides like glycogen or acidic mucins. Scale bar = 100µm. (C, D) Lipid staining of the 18mo INF/AMP region (N=3). Cytoplasmic vacuoles were negative for Oil Red O (C) and LipidTox Green (D), indicating that vacuoles did not contain lipid. (E-G) ATP1A1 IHC staining in the AIJ (E), ISM (F) and UTJ (G) regions isolated from 7mo mice, showing no specific localization to lateral surfaces or in the cytoplasm (N=3). (H) ATP1A1 IHC staining in the INF/AMP region isolated from 3mo mice, showing occasional punctate staining (N=3). Scale bar = 20µm.

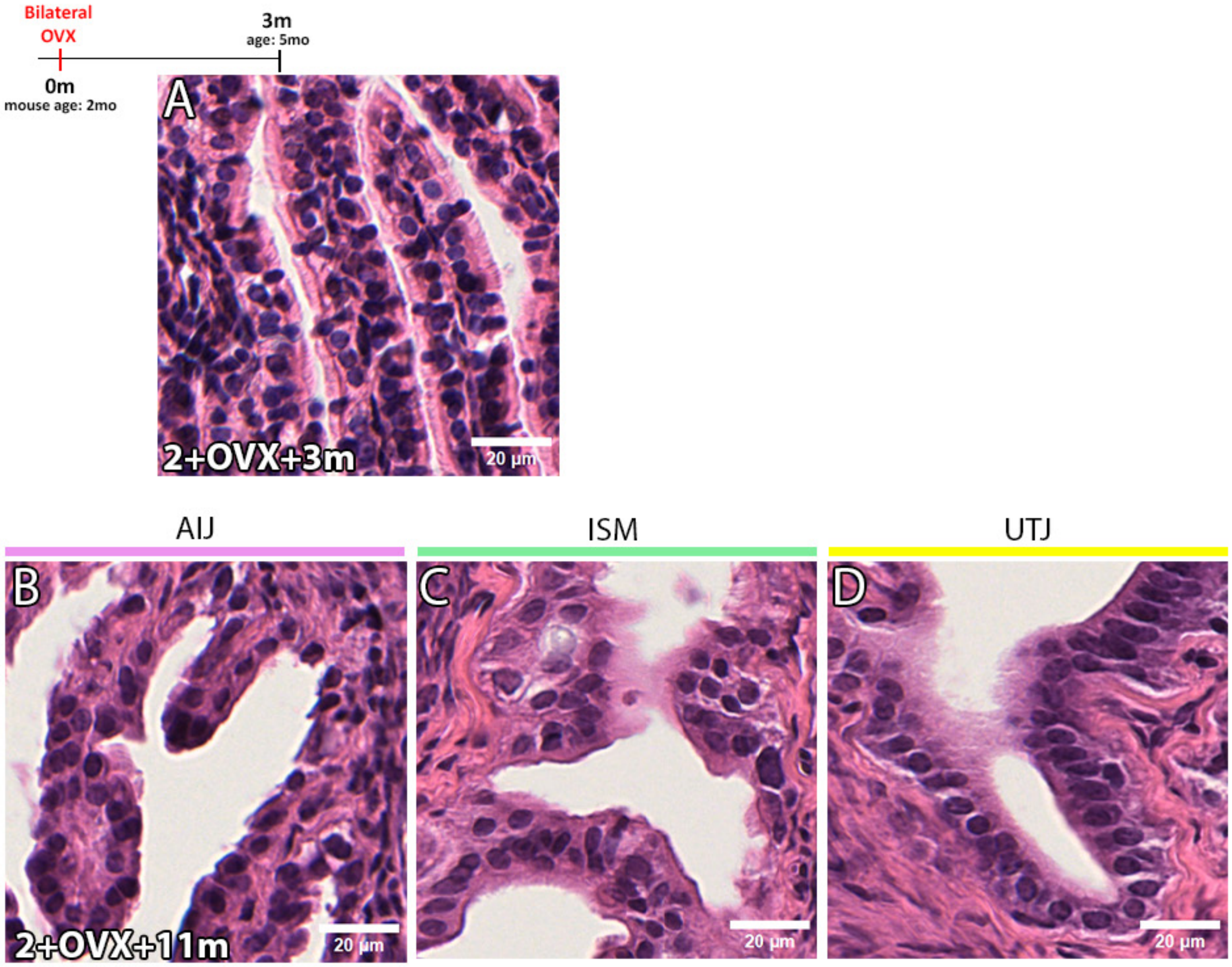

Suppl. Fig. 2: No discernible vacuoles in the INF/AMP region of mice 3 months following OVX and in the AIJ, ISM, and UTJ regions of mice 11 months post OVX. (A) H&E staining of the INF/AMP region in oviducts from mice 3 months post bilateral OVX (N=3). No discernible cytoplasmic vacuoles were noted. (B-D) H&E staining of the AIJ (B), ISM (C) and UTJ (D) regions in oviducts from mice 11 months post bilateral OVX (N=4). No discernible cytoplasmic vacuoles were noted. Scale bar = 20μm.

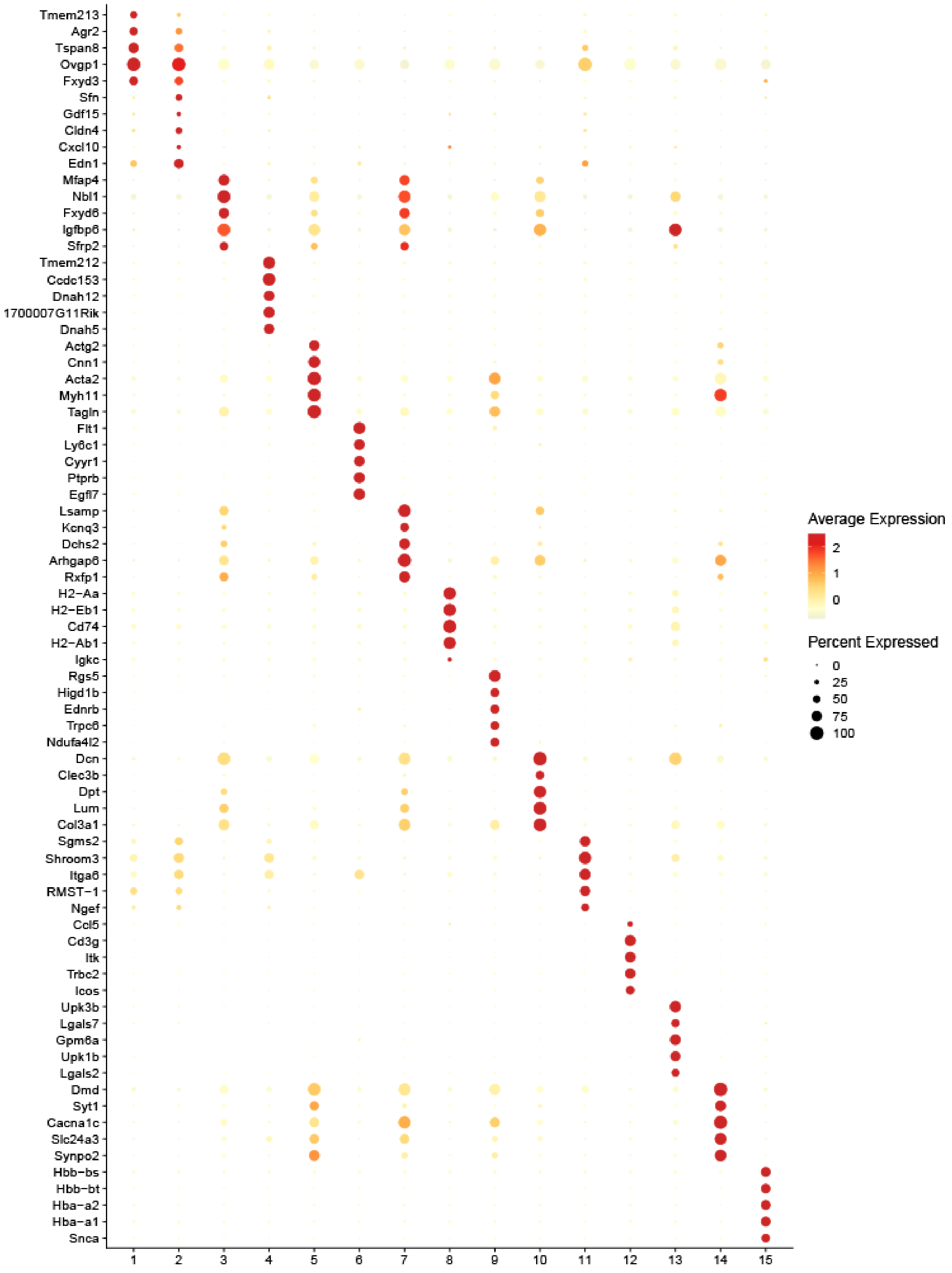

Suppl. Fig. 3: Dotplot of top 5 markers in each cluster.

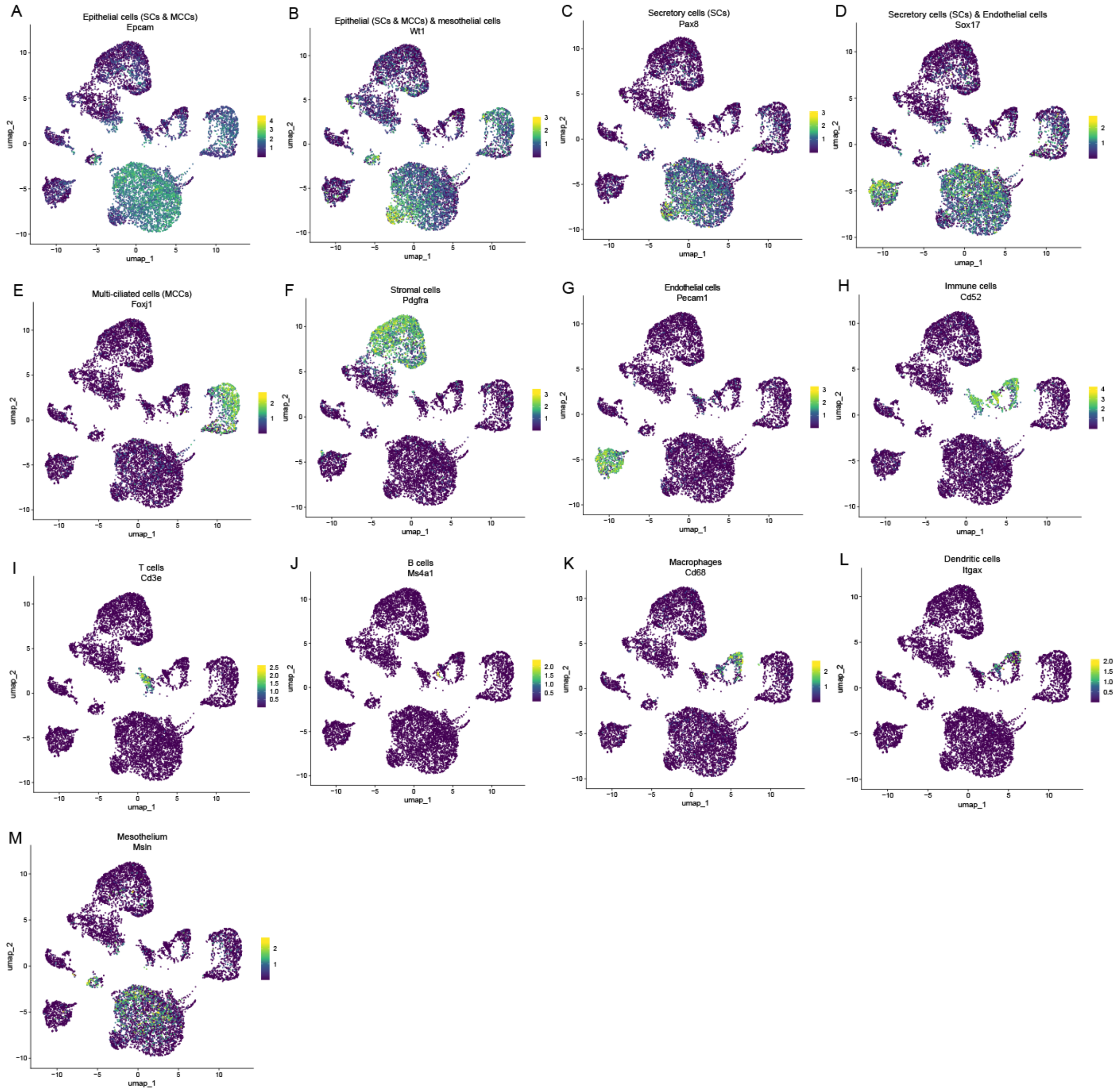

Suppl. Fig. 4: Identification of isolated cell populations using gene expression of known markers. (A-M) Gene expression of known markers that were not included in the dotplot, such as Epcam (A), Wt1 (B), Pax8 (C), Sox17 (D), Foxj1 (E), Pdgfra (F), Pecam1 (G), Cd52 (H), Cd3e (I), Cd20 (J), Cd68 (K), Cd11c (L), Msln (M).

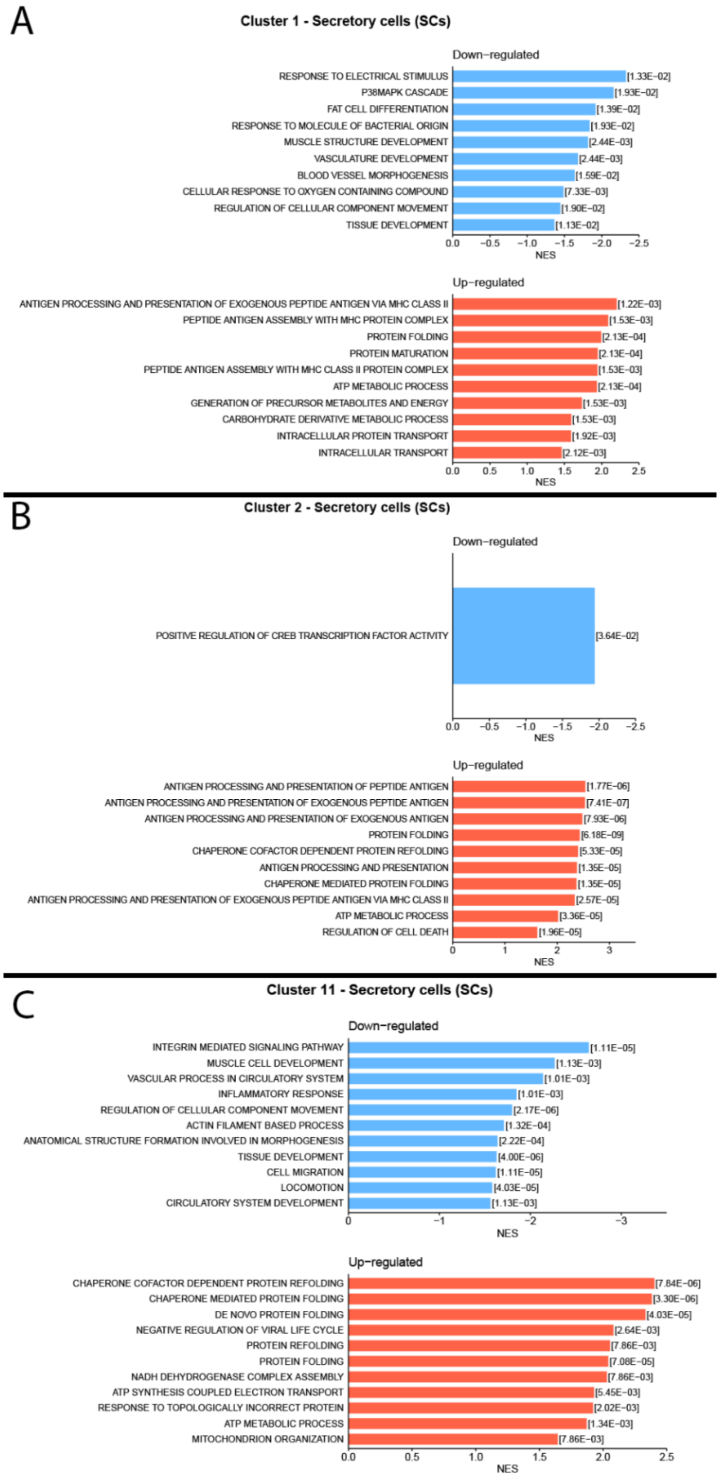

Suppl. Fig. 5: GSEA showing genesets down- or upregulated in each cluster of 18mo INF/AMP SCs, relative to 3mo INF/AMP SCs. (A-C) GSEA showing down- and upregulated genesets in SC clusters 1 (A), 2 (B), and 11 (C).

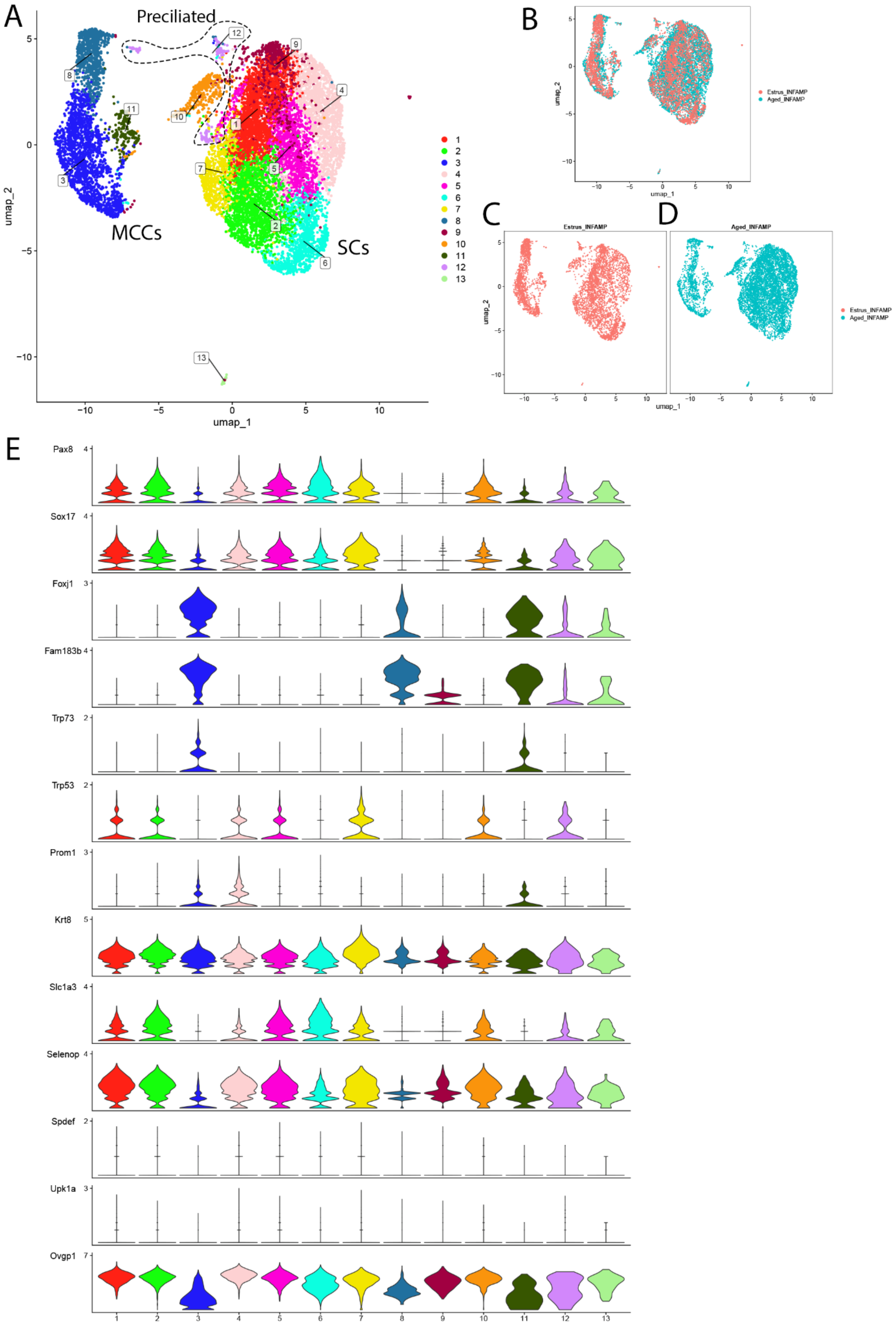

A
Preciliated
MCCs
SCs
umap_1
umap_2
B
Estrus_INFAMP
Aged_INFAMP
C
D
E
Pax8
Sox17
Foxj1
Fam183b
Trp73
Trp53
Prom1
Krt8
Slc1a3
Selenop
Spdef
Upk1a
Ovgp1

Suppl. Fig. 6: Identification of epithelial cell clusters using known markers. (A) Integrated UMAP plot of epithelial clusters 1, 2, 4, and 11 colour-coded by cluster membership. (B-D) UMAP clustering of epithelial cells from 3mo estrus stage mouse (C), 18mo mice (D), and overlap between the two (B) showing no significant alteration in clusters/cell states. (E) Violin plots showing expression of known INF/AMP SC and/or MCC markers among epithelial clusters.

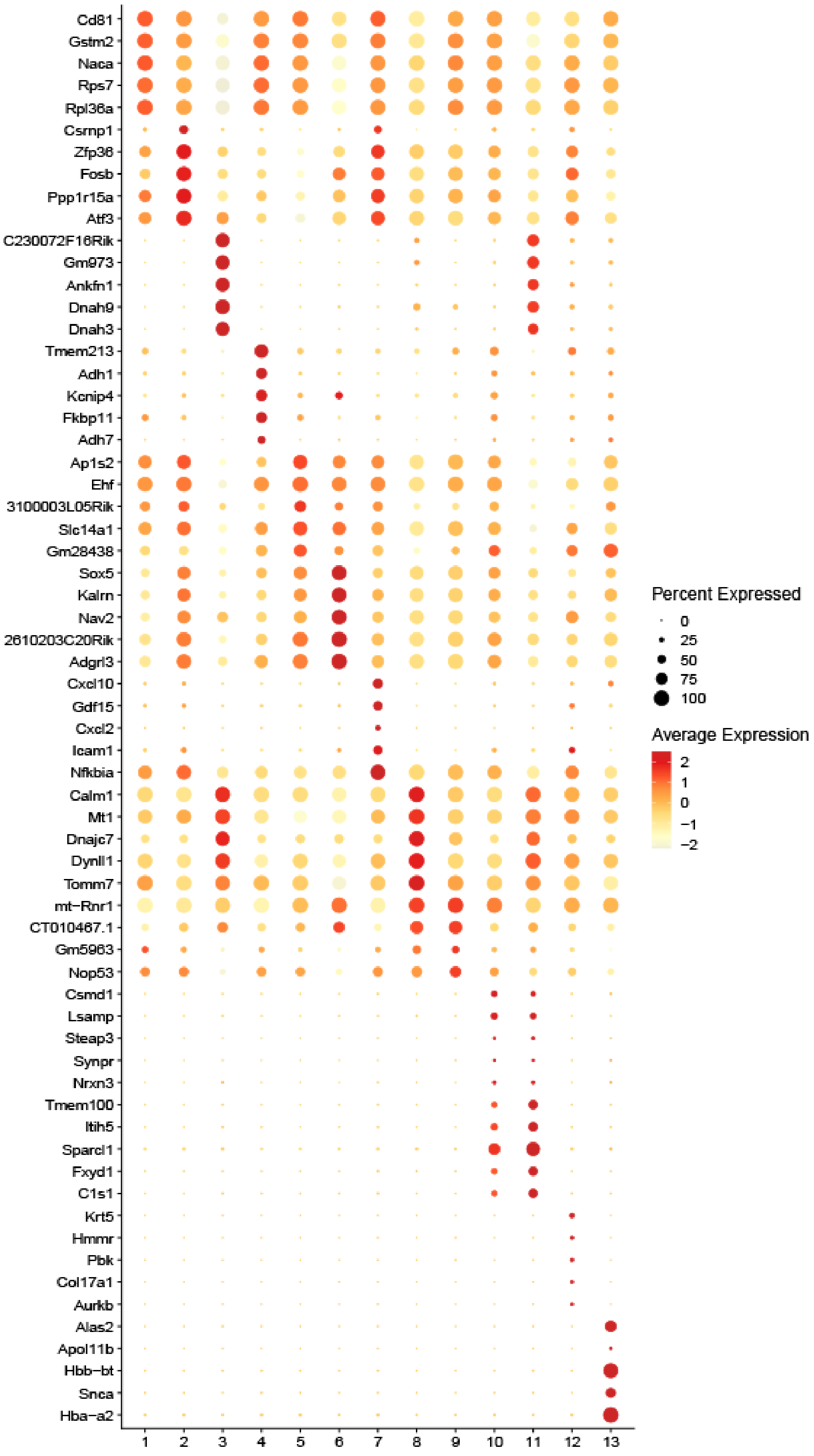

Suppl. Fig. 7: Dotplot of top 5 markers in each epithelial cluster.

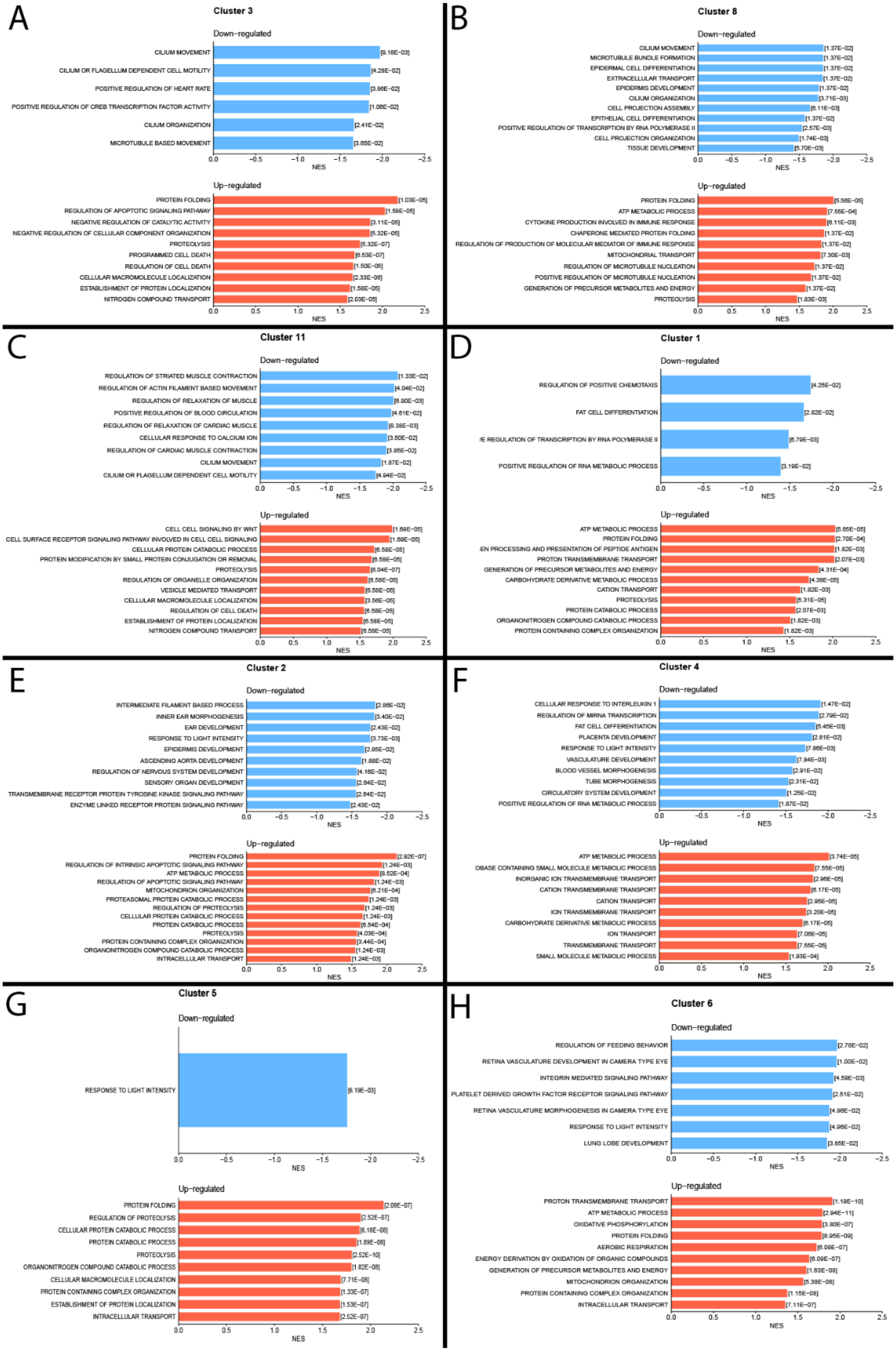
A
Cluster 3
Down−regulated
CILIUM MOVEMENT [9.18E−03]
CILIUM OR FLAGELLUM DEPENDENT CELL MOTILITY [4.29E−02]
POSITIVE REGULATION OF HEART RATE [3.96E−02]
POSITIVE REGULATION OF CREB TRANSCRIPTION FACTOR ACTIVITY [1.06E−02]
CILIUM ORGANIZATION [2.41E−02]
MICROTUBULE BASED MOVEMENT [3.65E−02]
NES
Up−regulated
PROTEIN FOLDING [1.03E−05]
REGULATION OF APOPTOTIC SIGNALING PATHWAY [1.58E−05]
NEGATIVE REGULATION OF CATALYTIC ACTIVITY [3.11E−05]
NEGATIVE REGULATION OF CELLULAR COMPONENT ORGANIZATION [5.32E−05]
PROTEOLYSIS [5.32E−07]
PROGRAMMED CELL DEATH [6.53E−07]
REGULATION OF CELL DEATH [1.50E−05]
CELLULAR MACROMOLECULE LOCALIZATION [2.33E−06]
ESTABLISHMENT OF PROTEIN LOCALIZATION [1.58E−05]
NITROGEN COMPOUND TRANSPORT [2.03E−05]
NES
B
Cluster 8
Down−regulated
CILIUM MOVEMENT [1.37E−02]
MICROTUBULE BUNDLE FORMATION [1.37E−02]
EPIDERMAL CELL DIFFERENTIATION [1.37E−02]
EXTRACELLULAR TRANSPORT [1.37E−02]
EPIDERMIS DEVELOPMENT [1.37E−02]
CILIUM ORGANIZATION [3.71E−03]
CELL PROJECTION ASSEMBLY [6.11E−03]
EPITHELIAL CELL DIFFERENTIATION [1.37E−02]
POSITIVE REGULATION OF TRANSCRIPTION BY RNA POLYMERASE II [2.57E−03]
CELL PROJECTION ORGANIZATION [1.74E−03]
TISSUE DEVELOPMENT [5.70E−03]
NES
Up−regulated
PROTEIN FOLDING [5.58E−05]
ATP METABOLIC PROCESS [7.55E−04]
CYTOKINE PRODUCTION INVOLVED IN IMMUNE RESPONSE [8.11E−03]
CHAPERONE MEDIATED PROTEIN FOLDING [1.37E−02]
REGULATION OF PRODUCTION OF MOLECULAR MEDIATOR OF IMMUNE RESPONSE [1.37E−02]
MITOCHONDRIAL TRANSPORT [7.30E−03]
REGULATION OF MICROTUBULE NUCLEATION [1.37E−02]
POSITIVE REGULATION OF MICROTUBULE NUCLEATION [1.37E−02]
GENERATION OF PRECURSOR METABOLITES AND ENERGY [1.37E−02]
PROTEOLYSIS [1.83E−03]
NES
C
Cluster 11
Down−regulated
REGULATION OF STRIATED MUSCLE CONTRACTION [1.33E−02]
REGULATION OF ACTIN FILAMENT BASED MOVEMENT [4.04E−02]
REGULATION OF RELAXATION OF MUSCLE [6.90E−03]
POSITIVE REGULATION OF BLOOD CIRCULATION [4.61E−02]
REGULATION OF RELAXATION OF CARDIAC MUSCLE [9.38E−03]
CELLULAR RESPONSE TO CALCIUM ION [3.50E−02]
REGULATION OF CARDIAC MUSCLE CONTRACTION [3.85E−02]
CILIUM MOVEMENT [1.87E−02]
CILIUM OR FLAGELLUM DEPENDENT CELL MOTILITY [4.94E−02]
NES
Up−regulated
CELL CELL SIGNALING BY WNT [1.69E−05]
CELL SURFACE RECEPTOR SIGNALING PATHWAY INVOLVED IN CELL CELL SIGNALING [1.69E−05]
CELLULAR PROTEIN CATABOLIC PROCESS [6.58E−05]
PROTEIN MODIFICATION BY SMALL PROTEIN CONJUGATION OR REMOVAL [6.58E−05]
PROTEOLYSIS [8.04E−07]
REGULATION OF ORGANELLE ORGANIZATION [6.58E−05]
VESICLE MEDIATED TRANSPORT [6.58E−05]
CELLULAR MACROMOLECULE LOCALIZATION [3.58E−05]
REGULATION OF CELL DEATH [6.58E−05]
ESTABLISHMENT OF PROTEIN LOCALIZATION [6.58E−05]
NITROGEN COMPOUND TRANSPORT [6.58E−05]
NES
D
Cluster 1
Down−regulated
REGULATION OF POSITIVE CHEMOTAXIS [4.25E−02]
FAT CELL DIFFERENTIATION [2.82E−02]
/E REGULATION OF TRANSCRIPTION BY RNA POLYMERASE II [6.79E−03]
POSITIVE REGULATION OF RNA METABOLIC PROCESS [3.19E−02]
NES
Up−regulated
ATP METABOLIC PROCESS [5.65E−05]
PROTEIN FOLDING [2.70E−04]
EN PROCESSING AND PRESENTATION OF PEPTIDE ANTIGEN [1.82E−03]
PROTON TRANSMEMBRANE TRANSPORT [2.07E−03]
GENERATION OF PRECURSOR METABOLITES AND ENERGY [4.31E−04]
CARBOHYDRATE DERIVATIVE METABOLIC PROCESS [4.38E−05]
CATION TRANSPORT [1.82E−03]
PROTEOLYSIS [5.31E−05]
PROTEIN CATABOLIC PROCESS [2.07E−03]
ORGANONITROGEN COMPOUND CATABOLIC PROCESS [1.82E−03]
PROTEIN CONTAINING COMPLEX ORGANIZATION [1.82E−03]
NES
E
Cluster 2
Down−regulated
INTERMEDIATE FILAMENT BASED PROCESS [2.86E−02]
INNER EAR MORPHOGENESIS [3.40E−02]
EAR DEVELOPMENT [2.43E−02]
RESPONSE TO LIGHT INTENSITY [3.73E−03]
EPIDERMIS DEVELOPMENT [2.85E−02]
ASCENDING AORTA DEVELOPMENT [1.88E−02]
REGULATION OF NERVOUS SYSTEM DEVELOPMENT [4.16E−02]
SENSORY ORGAN DEVELOPMENT [2.64E−02]
TRANSMEMBRANE RECEPTOR PROTEIN TYROSINE KINASE SIGNALING PATHWAY [2.64E−02]
ENZYME LINKED RECEPTOR PROTEIN SIGNALING PATHWAY [2.43E−02]
NES
Up−regulated
PROTEIN FOLDING [2.92E−07]
REGULATION OF INTRINSIC APOPTOTIC SIGNALING PATHWAY [1.24E−03]
ATP METABOLIC PROCESS [8.52E−04]
REGULATION OF APOPTOTIC SIGNALING PATHWAY [1.24E−03]
MITOCHONDRION ORGANIZATION [6.21E−04]
PROTEASOMAL PROTEIN CATABOLIC PROCESS [1.24E−03]
REGULATION OF PROTEOLYSIS [1.24E−03]
CELLULAR PROTEIN CATABOLIC PROCESS [1.24E−03]
PROTEIN CATABOLIC PROCESS [6.54E−04]
PROTEOLYSIS [4.03E−04]
PROTEIN CONTAINING COMPLEX ORGANIZATION [3.44E−04]
ORGANONITROGEN COMPOUND CATABOLIC PROCESS [1.24E−03]
INTRACELLULAR TRANSPORT [1.24E−03]
NES
F
Cluster 4
Down−regulated
CELLULAR RESPONSE TO INTERLEUKIN 1 [1.47E−02]
REGULATION OF MIRNA TRANSCRIPTION [2.79E−02]
FAT CELL DIFFERENTIATION [5.45E−03]
PLACENTA DEVELOPMENT [2.81E−02]
RESPONSE TO LIGHT INTENSITY [7.86E−03]
VASCULATURE DEVELOPMENT [7.84E−03]
BLOOD VESSEL MORPHOGENESIS [2.91E−02]
TUBE MORPHOGENESIS [2.31E−02]
CIRCULATORY SYSTEM DEVELOPMENT [1.25E−02]
POSITIVE REGULATION OF RNA METABOLIC PROCESS [1.87E−02]
NES
Up−regulated
ATP METABOLIC PROCESS [3.74E−05]
OBASE CONTAINING SMALL MOLECULE METABOLIC PROCESS [7.55E−05]
INORGANIC ION TRANSMEMBRANE TRANSPORT [2.96E−05]
CATION TRANSMEMBRANE TRANSPORT [6.17E−05]
CATION TRANSPORT [2.95E−05]
ION TRANSMEMBRANE TRANSPORT [3.20E−05]
CARBOHYDRATE DERIVATIVE METABOLIC PROCESS [6.17E−05]
ION TRANSPORT [7.06E−05]
TRANSMEMBRANE TRANSPORT [7.55E−05]
SMALL MOLECULE METABOLIC PROCESS [1.93E−04]
NES
G
Cluster 5
Down−regulated
RESPONSE TO LIGHT INTENSITY [6.19E−03]
NES
Up−regulated
PROTEIN FOLDING [2.08E−07]
REGULATION OF PROTEOLYSIS [2.52E−07]
CELLULAR PROTEIN CATABOLIC PROCESS [6.18E−08]
PROTEIN CATABOLIC PROCESS [1.89E−08]
PROTEOLYSIS [2.52E−10]
ORGANONITROGEN COMPOUND CATABOLIC PROCESS [1.82E−08]
CELLULAR MACROMOLECULE LOCALIZATION [7.71E−08]
PROTEIN CONTAINING COMPLEX ORGANIZATION [1.33E−07]
ESTABLISHMENT OF PROTEIN LOCALIZATION [1.53E−07]
INTRACELLULAR TRANSPORT [2.52E−07]
NES
H
Cluster 6
Down−regulated
REGULATION OF FEEDING BEHAVIOR [2.78E−02]
RETINA VASCULATURE DEVELOPMENT IN CAMERA TYPE EYE [1.00E−02]
INTEGRIN MEDIATED SIGNALING PATHWAY [4.59E−03]
PLATELET DERIVED GROWTH FACTOR RECEPTOR SIGNALING PATHWAY [2.51E−02]
RETINA VASCULATURE MORPHOGENESIS IN CAMERA TYPE EYE [4.96E−02]
RESPONSE TO LIGHT INTENSITY [4.96E−02]
LUNG LOBE DEVELOPMENT [3.85E−02]
NES
Up−regulated
PROTON TRANSMEMBRANE TRANSPORT [1.19E−10]
ATP METABOLIC PROCESS [2.94E−11]
OXIDATIVE PHOSPHORYLATION [3.80E−07]
PROTEIN FOLDING [8.95E−09]
AEROBIC RESPIRATION [6.09E−07]
ENERGY DERIVATION BY OXIDATION OF ORGANIC COMPOUNDS [6.09E−07]
GENERATION OF PRECURSOR METABOLITES AND ENERGY [1.63E−08]
MITOCHONDRION ORGANIZATION [5.38E−08]
PROTEIN CONTAINING COMPLEX ORGANIZATION [1.15E−08]
INTRACELLULAR TRANSPORT [7.11E−07]
NES

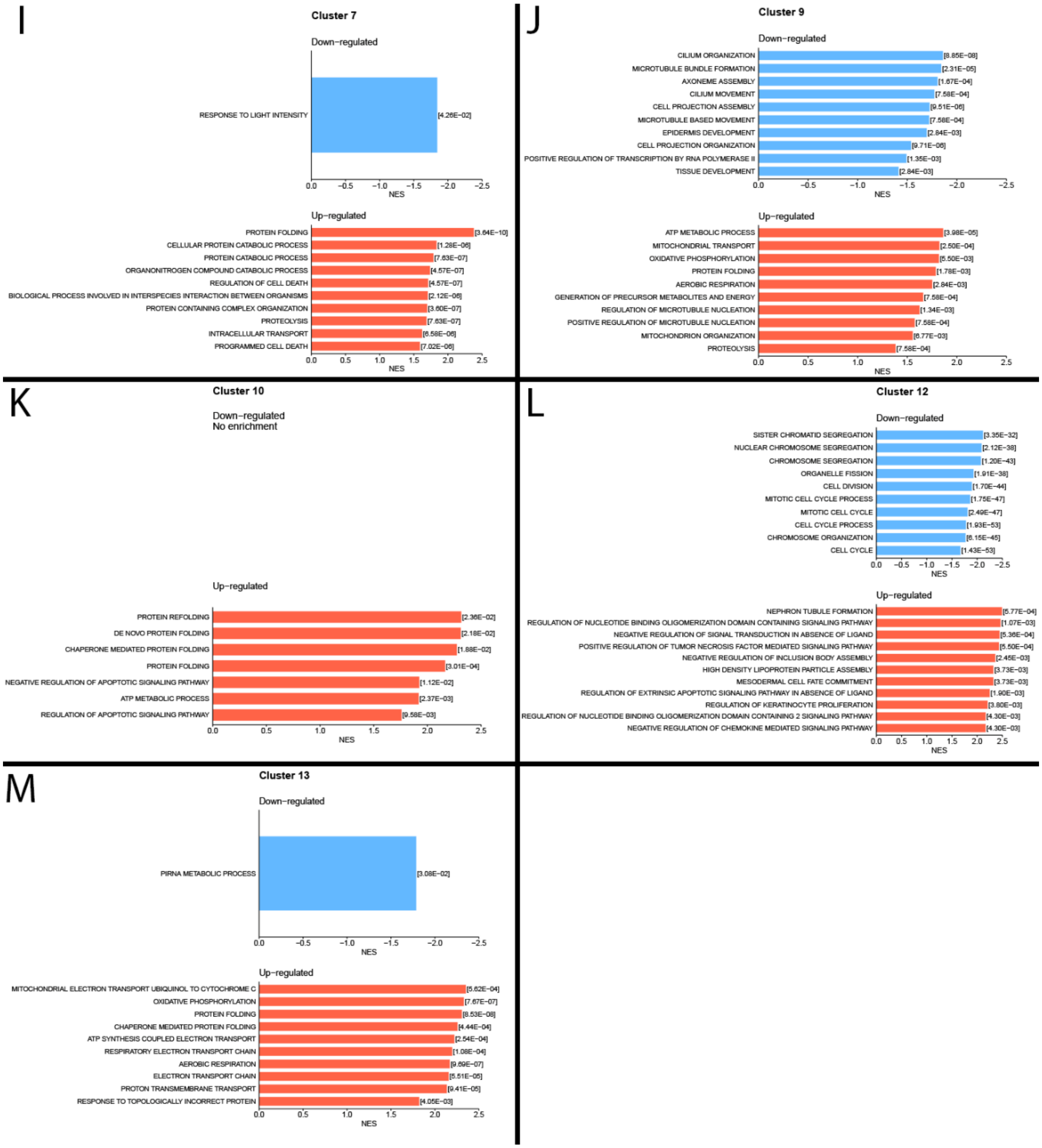

Suppl. Fig. 8: GSEA showing genesets down- or upregulated in 18mo INF/AMP epithelial cells, relative to 3mo INF/AMP epithelial cells. (A-C) GSEA showing down- and upregulated genesets in multi-ciliated cell clusters 3 (A), 8 (B), and 11 (C). (D-M) GSEA showing down- and upregulated genesets in secretory cell clusters 1 (D), 2 (E), 4-7 (F-I), and 9 (J), 10 (K), 12 (L), and 13 (M).

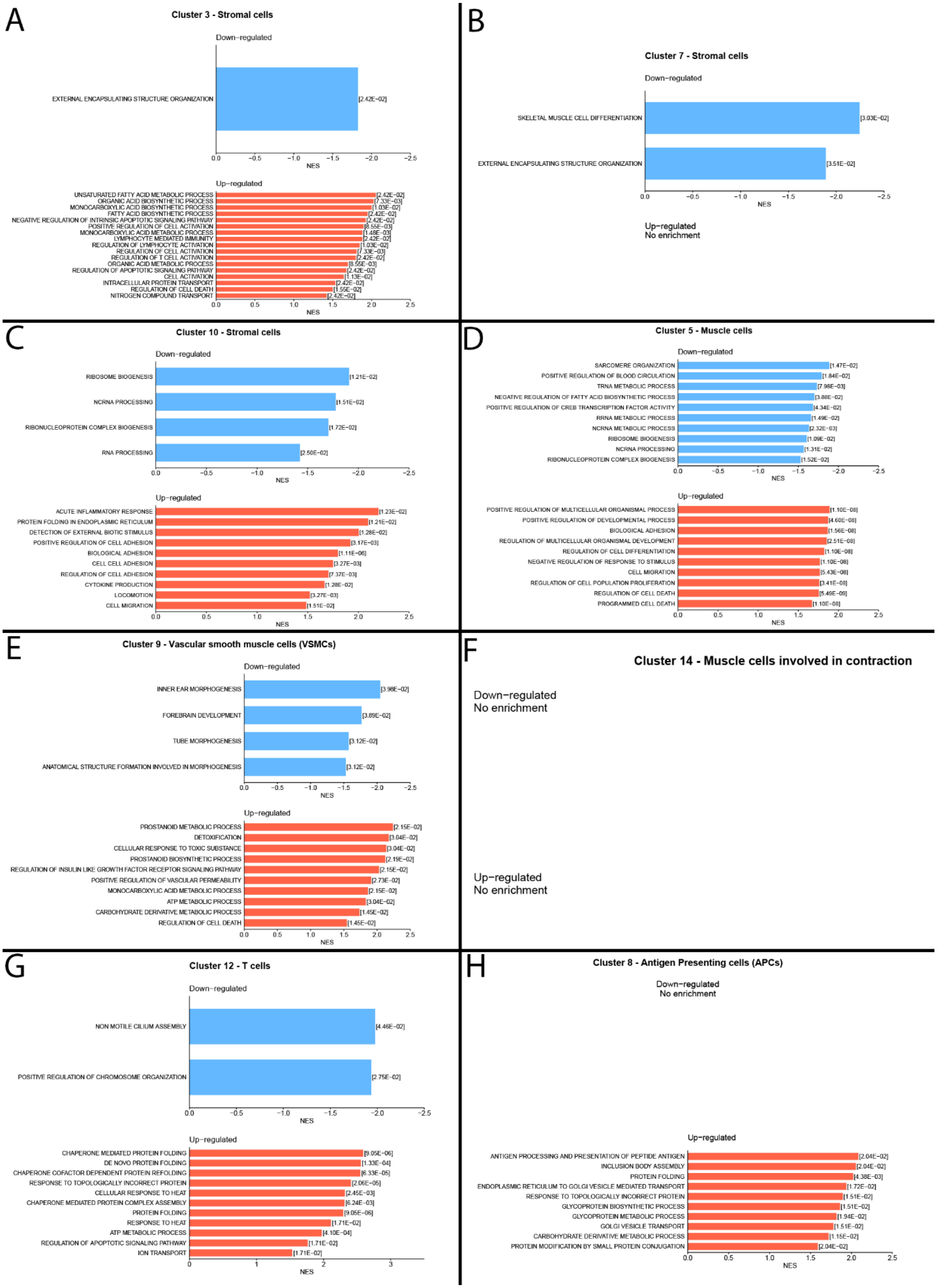
A
Cluster 3 - Stromal cells
Down−regulated
EXTERNAL ENCAPSULATING STRUCTURE ORGANIZATION [2.42E−02]
0.0 −0.5 −1.0 −1.5 −2.0 −2.5
NES
Up−regulated
UNSATURATED FATTY ACID METABOLIC PROCESS [2.42E−02]
ORGANIC ACID BIOSYNTHETIC PROCESS [7.33E−03]
MONOCARBOXYLIC ACID BIOSYNTHETIC PROCESS [1.03E−02]
FATTY ACID BIOSYNTHETIC PROCESS [2.42E−02]
NEGATIVE REGULATION OF INTRINSIC APOPTOTIC SIGNALING PATHWAY [2.42E−02]
POSITIVE REGULATION OF CELL ACTIVATION [8.55E−03]
MONOCARBOXYLIC ACID METABOLIC PROCESS [1.48E−03]
LYMPHOCYTE MEDIATED IMMUNITY [2.42E−02]
REGULATION OF LYMPHOCYTE ACTIVATION [1.03E−02]
REGULATION OF CELL ACTIVATION [7.33E−03]
REGULATION OF T CELL ACTIVATION [2.42E−02]
ORGANIC ACID METABOLIC PROCESS [8.55E−03]
REGULATION OF APOPTOTIC SIGNALING PATHWAY [2.42E−02]
CELL ACTIVATION [1.13E−02]
INTRACELLULAR PROTEIN TRANSPORT [2.42E−02]
REGULATION OF CELL DEATH [1.55E−02]
NITROGEN COMPOUND TRANSPORT [2.42E−02]
0.0 0.5 1.0 1.5 2.0 2.5
NES
B
Cluster 7 - Stromal cells
Down−regulated
SKELETAL MUSCLE CELL DIFFERENTIATION [3.03E−02]
EXTERNAL ENCAPSULATING STRUCTURE ORGANIZATION [3.51E−02]
0.0 −0.5 −1.0 −1.5 −2.0 −2.5
NES
Up−regulated
No enrichment
C
Cluster 10 - Stromal cells
Down−regulated
RIBOSOME BIOGENESIS [1.21E−02]
NCRNA PROCESSING [1.51E−02]
RIBONUCLEOPROTEIN COMPLEX BIOGENESIS [1.72E−02]
RNA PROCESSING [2.50E−02]
0.0 −0.5 −1.0 −1.5 −2.0 −2.5
NES
Up−regulated
ACUTE INFLAMMATORY RESPONSE [1.23E−02]
PROTEIN FOLDING IN ENDOPLASMIC RETICULUM [1.21E−02]
DETECTION OF EXTERNAL BIOTIC STIMULUS [1.28E−02]
POSITIVE REGULATION OF CELL ADHESION [3.17E−03]
BIOLOGICAL ADHESION [1.11E−06]
CELL CELL ADHESION [3.27E−03]
REGULATION OF CELL ADHESION [7.37E−03]
CYTOKINE PRODUCTION [1.28E−02]
LOCOMOTION [3.27E−03]
CELL MIGRATION [1.51E−02]
0.0 0.5 1.0 1.5 2.0 2.5
NES
D
Cluster 5 - Muscle cells
Down−regulated
SARCOMERE ORGANIZATION [1.47E−02]
POSITIVE REGULATION OF BLOOD CIRCULATION [1.84E−02]
TRNA METABOLIC PROCESS [7.98E−03]
NEGATIVE REGULATION OF FATTY ACID BIOSYNTHETIC PROCESS [3.88E−02]
POSITIVE REGULATION OF CREB TRANSCRIPTION FACTOR ACTIVITY [4.34E−02]
RRNA METABOLIC PROCESS [1.49E−02]
NCRNA METABOLIC PROCESS [2.32E−03]
RIBOSOME BIOGENESIS [1.09E−02]
NCRNA PROCESSING [1.31E−02]
RIBONUCLEOPROTEIN COMPLEX BIOGENESIS [1.52E−02]
0.0 −0.5 −1.0 −1.5 −2.0 −2.5
NES
Up−regulated
POSITIVE REGULATION OF MULTICELLULAR ORGANISMAL PROCESS [1.10E−08]
POSITIVE REGULATION OF DEVELOPMENTAL PROCESS [4.60E−08]
BIOLOGICAL ADHESION [1.56E−08]
REGULATION OF MULTICELLULAR ORGANISMAL DEVELOPMENT [2.51E−08]
REGULATION OF CELL DIFFERENTIATION [1.10E−08]
NEGATIVE REGULATION OF RESPONSE TO STIMULUS [1.10E−08]
CELL MIGRATION [5.43E−08]
REGULATION OF CELL POPULATION PROLIFERATION [3.41E−08]
REGULATION OF CELL DEATH [5.49E−09]
PROGRAMMED CELL DEATH [1.10E−08]
0.0 0.5 1.0 1.5 2.0 2.5
NES
E
Cluster 9 - Vascular smooth muscle cells (VSMCs)
Down−regulated
INNER EAR MORPHOGENESIS [3.98E−02]
FOREBRAIN DEVELOPMENT [3.89E−02]
TUBE MORPHOGENESIS [3.12E−02]
ANATOMICAL STRUCTURE FORMATION INVOLVED IN MORPHOGENESIS [3.12E−02]
0.0 −0.5 −1.0 −1.5 −2.0 −2.5
NES
Up−regulated
PROSTANOID METABOLIC PROCESS [2.15E−02]
DETOXIFICATION [3.04E−02]
CELLULAR RESPONSE TO TOXIC SUBSTANCE [3.04E−02]
PROSTANOID BIOSYNTHETIC PROCESS [2.19E−02]
REGULATION OF INSULIN LIKE GROWTH FACTOR RECEPTOR SIGNALING PATHWAY [2.15E−02]
POSITIVE REGULATION OF VASCULAR PERMEABILITY [2.73E−02]
MONOCARBOXYLIC ACID METABOLIC PROCESS [2.15E−02]
ATP METABOLIC PROCESS [3.04E−02]
CARBOHYDRATE DERIVATIVE METABOLIC PROCESS [1.45E−02]
REGULATION OF CELL DEATH [1.45E−02]
0.0 0.5 1.0 1.5 2.0 2.5
NES
F
Cluster 14 - Muscle cells involved in contraction
Down−regulated
No enrichment
Up−regulated
No enrichment
G
Cluster 12 - T cells
Down−regulated
NON MOTILE CILIUM ASSEMBLY [4.46E−02]
POSITIVE REGULATION OF CHROMOSOME ORGANIZATION [2.75E−02]
0.0 −0.5 −1.0 −1.5 −2.0 −2.5
NES
Up−regulated
CHAPERONE MEDIATED PROTEIN FOLDING [9.05E−06]
DE NOVO PROTEIN FOLDING [1.33E−04]
CHAPERONE COFACTOR DEPENDENT PROTEIN REFOLDING [6.33E−05]
RESPONSE TO TOPOLOGICALLY INCORRECT PROTEIN [2.06E−05]
CELLULAR RESPONSE TO HEAT [2.45E−03]
CHAPERONE MEDIATED PROTEIN COMPLEX ASSEMBLY [6.24E−03]
PROTEIN FOLDING [9.05E−06]
RESPONSE TO HEAT [1.71E−02]
ATP METABOLIC PROCESS [4.10E−04]
REGULATION OF APOPTOTIC SIGNALING PATHWAY [1.71E−02]
ION TRANSPORT [1.71E−02]
0 1 2 3
NES
H
Cluster 8 - Antigen Presenting cells (APCs)
Down−regulated
No enrichment
Up−regulated
ANTIGEN PROCESSING AND PRESENTATION OF PEPTIDE ANTIGEN [2.04E−02]
INCLUSION BODY ASSEMBLY [2.04E−02]
PROTEIN FOLDING [4.38E−03]
ENDOPLASMIC RETICULUM TO GOLGI VESICLE MEDIATED TRANSPORT [1.72E−02]
RESPONSE TO TOPOLOGICALLY INCORRECT PROTEIN [1.51E−02]
GLYCOPROTEIN BIOSYNTHETIC PROCESS [1.51E−02]
GLYCOPROTEIN METABOLIC PROCESS [1.94E−02]
GOLGI VESICLE TRANSPORT [1.51E−02]
CARBOHYDRATE DERIVATIVE METABOLIC PROCESS [1.15E−02]
PROTEIN MODIFICATION BY SMALL PROTEIN CONJUGATION [2.04E−02]
0.0 0.5 1.0 1.5 2.0 2.5
NES

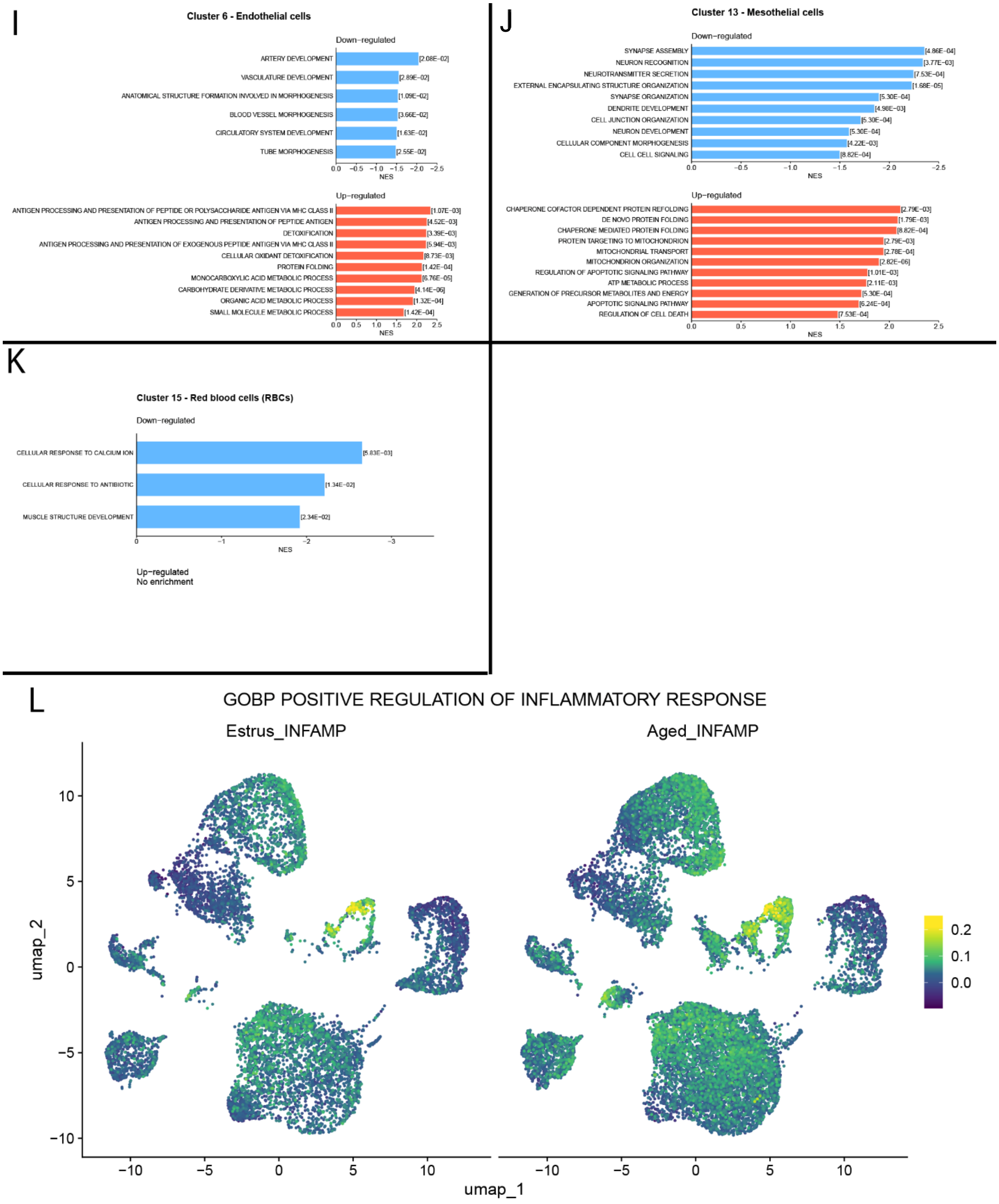

Suppl. Fig. 9: GSEA of each cluster showing genesets down- or upregulated in 18mo INF/AMP cells, relative to 3mo INF/AMP cells. (A-C) GSEA showing down- and upregulated

genesets in stromal cell clusters 3 (A), 7 (B), and 10 (C). (D-F) GSEA showing down- and upregulated genesets in muscle cell clusters 5 (D), 9 (E), and 14 (F). (G, H) GSEA showing down- and upregulated genesets in immune cell clusters, including cluster 12, identified as T-cells (G), and cluster 8, identified as antigen presenting cells/APCs (H). (I) GSEA showing down- and upregulated genesets in cluster 6, identified as endothelial cells. (J) GSEA showing down- and upregulated genesets in cluster 13, identified as mesothelial cells. (K) GSEA showing down- and upregulated genesets in cluster 15, identified as red blood cells/RBCs. (L) UMAP showing expression of gene set associated with positive regulation of inflammatory response in young (left) and aged (right) INF/AMP cells.

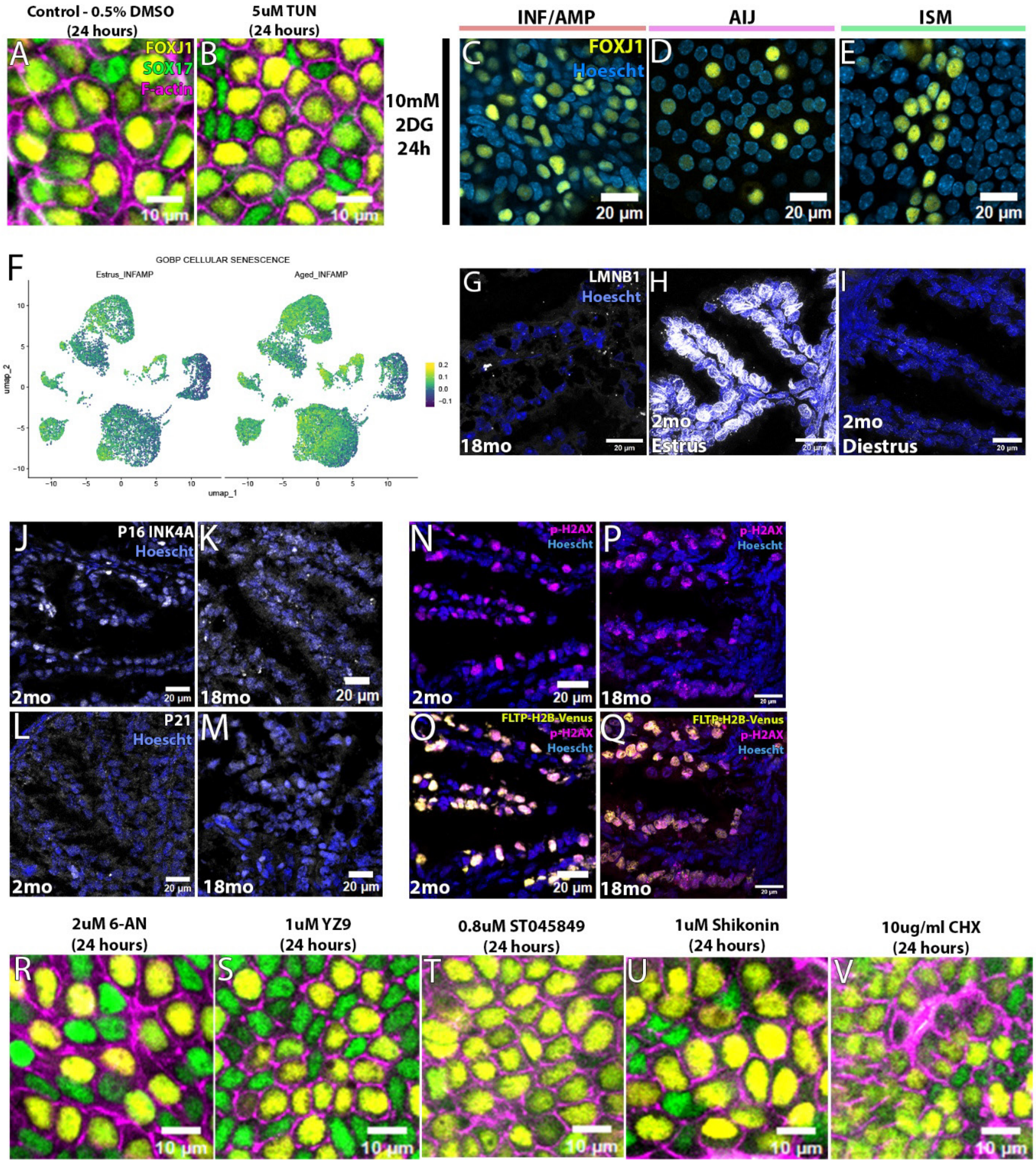

Suppl. Fig. 10: ER stress, inhibition of glycolysis, pentose phosphate, or hexosamine pathways in *in vitro* organotypic slice cultures did not result in discernible vacuolation. (A, B) ER stress was induced by treating with Tunicamycin/TUN for 24 hours in INF/AMP

organotypic slice cultures. No obvious cellular differences were noted in treated cultures (B) and controls (A); N=3. Scale bar = 10µm. (C-E) Inhibition of glycolysis in organotypic slice cultures. 24-hour-long 2DG treatment did not induce any obvious cellular differences in the INF/AMP (C), AIJ (D), or ISM (E); N=3. Scale bar = 20µm. (F-K) Cellular senescence markers in the INF/AMP region of aged and young mice. Expression of cellular senescence-associated geneset between young (left) and aged (right) INF/AMP cells (F). Lamin B1 was not detected in 18mo INF/AMP (G). Distinct LaminB1 expression levels in the INF/AMP region of 2mo mice in estrus (H) and diestrus stages (I). Similar expression patterns of P16 (J, K), P21 (L, M) and phospho-H2AX (N-Q) in young and aged mice. Phospho-H2AX is expressed in INF/AMP MCCs of young (N, O) and aged mice (P, Q). Scale bar = 20µm. (R-V) Induction of metabolic stress/nutrient deprivation in INF/AMP slice cultures. No cellular differences were noted upon inhibition of pentose phosphate pathway (R), PFKFB3 (S), hexosamine biosynthesis pathway (T), PKM2 (U), and protein synthesis (V); N=3. Scale bar = 10µm.

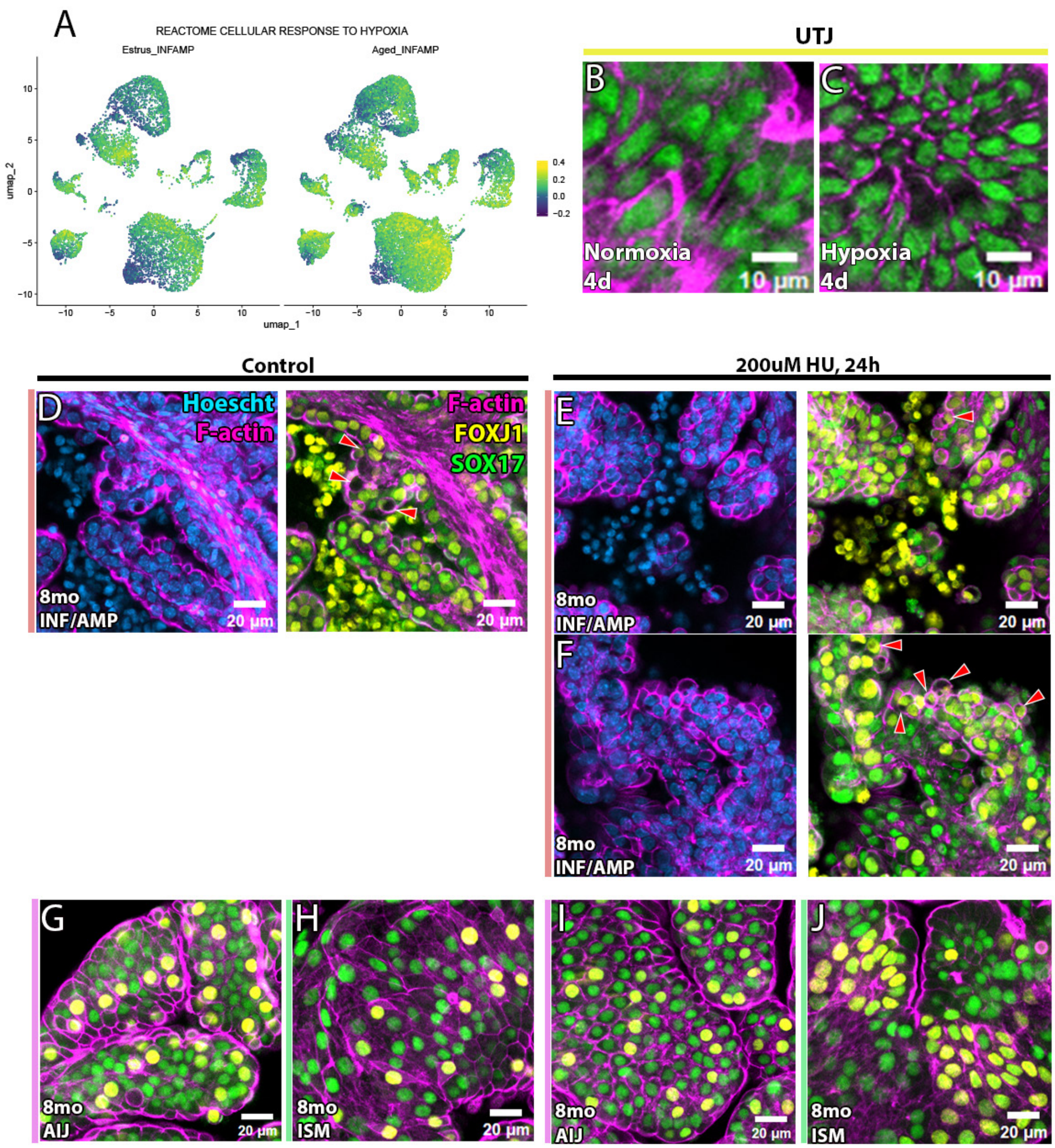

Suppl. Fig. 11: Low dose HU treatment in *in vitro* organotypic slice cultures from 8mo mice did not result in increased MCC vacuolation. (A) UMAP showing expression of geneset associated with cellular response to hypoxia in young (left) and aged (right) INF/AMP cells. (B, C) No cellular changes in UTJ epithelium in normoxia (B; N=3) and hypoxia (C); N=3. Scale bar

= 10µm. (D-F) Low dose HU treatment following *in vitro* organotypic culture of slices from 8mo mice did not result in discernible differences, as compared to the control. Condensed, protruding nuclei and vacuolated MCCs (red arrows) were noted in both control (D) and 200µM HU treated (E, F) INF/AMP slices from 8mo mice (N=3). (G-J) No changes in cellular morphology were noted in control and treated AIJ (G, I) or ISM (H, J) slices from 8mo mice (N=3). Scale bar = 20µm.

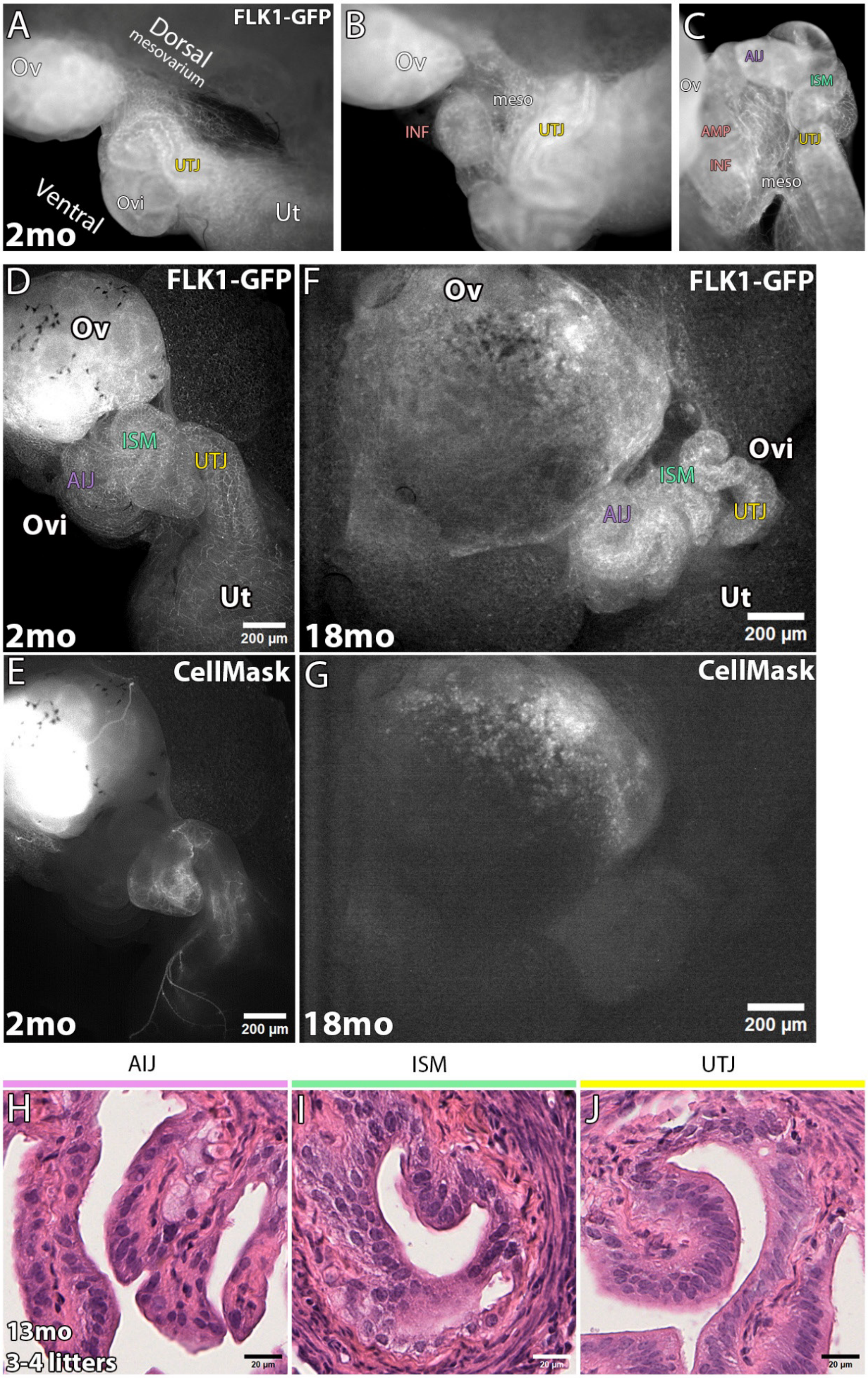

A
FLK1-GFP
Dorsal
mesovarium
Ov
UTJ
Ventral
Ovi
Ut
2mo
B
Ov
meso
INF
UTJ
C
AIJ
Ov
ISM
AMP
UTJ
INF
meso
D
FLK1-GFP
Ov
ISM
UTJ
AIJ
Ovi
Ut
2mo
200 µm
F
FLK1-GFP
Ov
Ovi
ISM
AIJ
UTJ
Ut
18mo
200 µm
E
CellMask
2mo
200 µm
G
CellMask
18mo
200 µm
AIJ
ISM
UTJ
H
13mo
3-4 litters
20 µm
I
20 µm
J
20 µm

Suppl. Fig. 12: No CellMask noted in perfused 18mo mice, and no discernible vacuoles in the AIJ, ISM, and UTJ regions of 13mo mice with 3-4 litters. (A-C) FLK1-GFP expressing vessels formed an intricate network throughout the upper reproductive tract, likely labeling arterioles and/or capillaries. Images correspond to Fig. 7C-E, N=3. (D-G) CellMask labeling was not observed in the aged upper female reproductive tract. Tracts isolated from 2mo (D) and 18mo (F) mice show FLK1-GFP+ vessels. No CellMask labeling noted in ovary and oviduct isolated from a perfused 18mo mouse (G; N=3), as compared to 2mo mice (E); N=3. Scale bar = 200µm. (H-J) Vacuoles were not reproducibly observed in the AIJ (H), ISM (I) and UTJ (J) regions of 13mo mice that had given birth to 3-4 litters (N=3). Scale bar = 20µm.

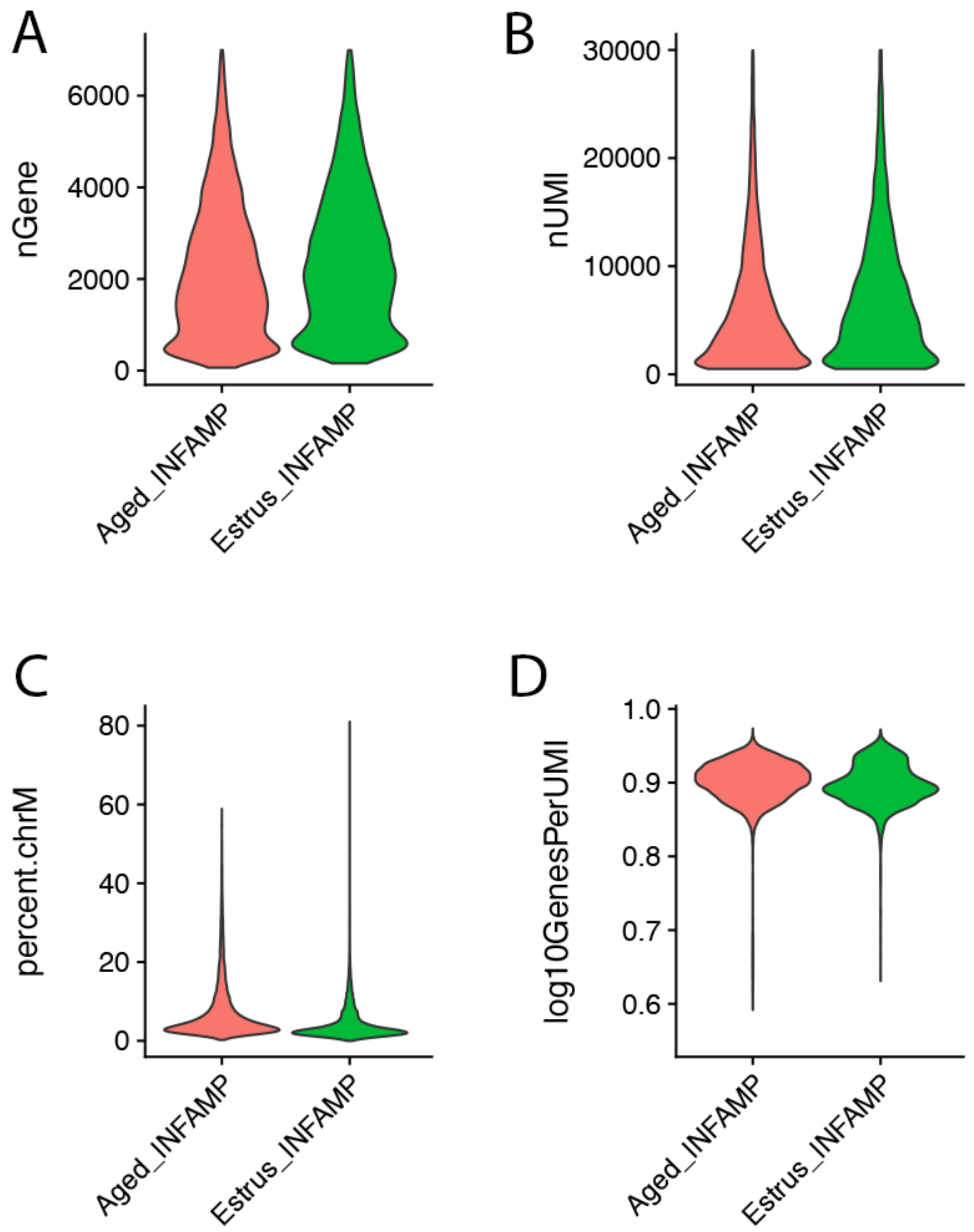

Suppl. Fig. 13: **Quality control metrics for scRNASeq datsets**. (A-C) nGene (A), nUMI (B), percent.chrM (C), and log10 genes per UMI (D) metrics in Aged_INFAMP (left) and Estrus_INFAMP (right) scRNASeq datasets.

Suppl. Vid. 1: Cilia beating continued even in aged mice. Brightfield video of cilia beating in the INF region of a 13mo mouse (50 frames per second; N=3).

Suppl. Vid. 2: Cilia beating in young mice. Brightfield video of cilia beating in the INF region of a 2mo mouse (50 frames per second; N=3).
